# ﻿Floristic diversity and assessment of the conservation status of Togo’s plant species

**DOI:** 10.3897/phytokeys.261.151951

**Published:** 2025-08-19

**Authors:** Folega Fousséni, Bawa Demirel Maza-esso, Atakpama Wouyo, Badjaré Bilouktime, Jasmina Šinžar-Sekulić, Wala Kperkouma, Batawila Komlan, Akpagana Koffi

**Affiliations:** 1 Laboratory of Botany and Plant Ecology, Department of Botany, Faculty of Sciences, University of Lomé, Lomé-Togo 01 BP 1515, Togo University of Lomé Lomé Togo; 2 University of Belgrade, Faculty of Biology, Institute of Botany and Botanical Garden “Jevremovac” Takovska 43, 11000 Belgrade, Serbia University of Belgrade Belgrade Serbia

**Keywords:** Biodiversity, conservation, flora, threatened species, Togo

## Abstract

Regular updates of floristic lists are essential for assessing the state of flora, identifying threatened species, and guiding conservation actions. This study aims to provide a comprehensive assessment of the floristic diversity and conservation status of plant species across selected ecological zones in Togo. A total of 121 plots, with areas determined based on the types of vegetation formations, were randomly selected from a systematic grid of points spaced 5 km apart, generated using QGIS 3.24.1 and overlaid on Togo’s map. A total of 498 plant species distributed across 337 genera and 92 families were recorded. The most represented families were Fabaceae (86 species) and Poaceae (48 species). Biological types were dominated by microphanerophytes (25.7%) and nanophanerophytes (18.67%), followed by therophytes (15.06%). Species from the Guineo-Congolian/Sudano-Zambesian transition zone (34.54%) were the most widespread, followed by Guineo-Congolian (23.09%) and Sudano-Zambesian (29.89%) regions. The distribution of species by ecological zones showed high diversity in Zone III. A total of 09 threatened species, 48 invasive and/or alien species, 31 agroforestry species, and 23 soil-fertility-enhancing species were identified. This study provides a rapid and updated understanding of the state of flora. The study emphasizes the urgency for ongoing floristic monitoring and the need for targeted conservation strategies to mitigate threats posed by anthropogenic pressures and invasive species. These findings provide a critical understanding of Togo’s flora, guiding effective conservation actions.

## ﻿Introduction

Changes in species composition are indicators of environmental changes such as deforestation, restoration, climate change, pollution, or other anthropogenic pressures (Phillips et al. 2023). Updating the floristic list of a region is essential for assessing and protecting plant biodiversity, providing a strong basis for conservation and sustainable ecosystem management (Uniyal and Singh 2014; Rana et al. 2020). It helps detect plant species that have undergone changes in their habitat, population, or conservation status, thereby prioritizing conservation efforts for threatened or endangered species.

Flora conservation in Togo, as in many countries, is a complex issue involving a balance between environmental preservation and socio-economic development (Fandjinou et al. 2020). Togo’s vegetation includes a diversity of ecosystems: savannas, forests, crops, and mangroves. Like many African countries, Togo faces challenges such as deforestation, habitat degradation due to unsustainable land use practices, and climate change impacts, which threaten its plant diversity (Wala et al. 2012a; Kombate et al. 2020). In response to these challenges, Togo has made efforts to protect its biodiversity through various conservation initiatives and policies (Dao et al. 2021; Adjossou et al. 2022b; MERF 2022). These include the creation of protected areas such as national parks and nature reserves to safeguard important habitats and species. Additionally, the country has engaged in international conservation agreements and initiatives to address transboundary conservation issues and promote sustainable land management practices.

Major challenges remain, including continuous monitoring of national flora and the variability of their status and the state of plant ecosystems. However, the last national assessment considering both woody and herbaceous species was conducted several years ago, making it difficult to assess the current state of flora and its evolution. Under the combined effects of anthropogenic pressures (deforestation, urbanization, extensive agriculture, bushfires) and climate change (changes in temperature and precipitation regimes), the structure and floristic composition of Togo’s ecosystems are undergoing significant transformations. These changes affect not only the distribution of native species but also favor the proliferation of invasive species, leading to increased ecological competition and potential extinction of vulnerable local species (Akodéwou et al. 2019). In this context, it becomes imperative to update knowledge on floristic diversity to identify evolutionary trends and the factors responsible for these changes. A rigorous assessment would enhance the understanding of ecological dynamics impacting flora, evaluate the representation of threatened species, and develop appropriate conservation strategies. Moreover, better knowledge of species distribution and conservation status would strengthen protected area management and propose specific actions to preserve the country’s plant wealth. This study thus contributes to updating floristic data by integrating an in-depth analysis of recorded species, their biogeographic distribution, and the threats they face.

This study aims to provide a targeted assessment of the floristic diversity and conservation status of selected ecological zones in Togo, thereby establishing a robust baseline for long-term monitoring and effective conservation strategies that address the impacts of anthropogenic pressures on plant species. Specifically, it seeks to:

- Conduct a targeted floristic inventory within selected ecological zones by establishing a baseline data for long-term monitoring.
- Analyze biological diversity based on biological and phytogeographic types to identify ecological and biogeographic trends.
- Assess the conservation status of identified species through classification according to the IUCN Red List and the presence of invasive species.

## ﻿Methods

### ﻿Study area

Togo is a country located in West Africa. It is bordered by Ghana to the west, Benin to the east, Burkina Faso to the north, and the Atlantic Ocean, specifically the Gulf of Guinea, to the south. The country extends approximately between 6°05.39'N and 11°07.00'N latitude and between 0°07.49'W and 1°47.26'E longitude, covering an area of about 56,600 square kilometers. It is divided into five administrative regions: Savanna, Kara, Centrale, Plateaux, and Maritime. Its population is estimated at 8,095,498 inhabitants, with a demographic growth rate of 2.3% and an urbanization rate of 42% (INSEED 2022).

**Figure 1.**
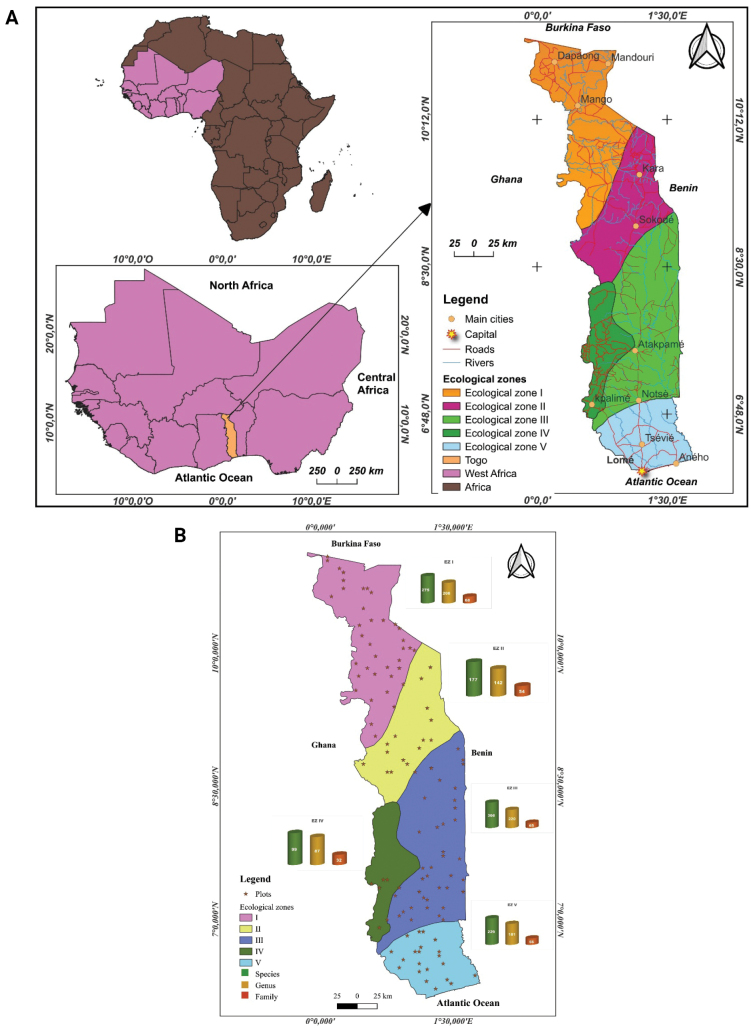
A. Location of the study area. Map showing the geographical location of Togo in West Africa, with major administrative regions and latitudinal and longitudinal boundaries. The ecological zones referenced in the study are overlaid, providing spatial context for the floristic inventory; B. Distribution of sampling plots and diversity of ecological zones. Map illustrating the spatial distribution of the 121 floristic sampling plots across Togo. The plots are stratified across five ecological zones (I–V), enabling the assessment of plant diversity in relation to climatic and ecological variation.

Togo has a Sudano-Guinean climate with two distinct climatic regimes: a tropical Guinean climate in the south and a tropical Sudanian climate in the north. The southern half experiences four seasons, with two rainy seasons from April to June and from September to October, and two dry seasons. The northern half experiences a rainy season from April to October and a dry season from November to March, with annual precipitation ranging from 800 to 1,200 mm and temperature variations between 18° and 38 °C. Ern (1979) defined Togo’s main phytoecological traits based on floristic and geomorphological data (Fig. 1A):

- Ecological zone I: The northern plains zone is an important agroforestry region dominated by agroforestry parks containing various tree species such a *Vitellariaparadoxa*, *Borassusaethiopum*, *Parkiabiglobosa*, *Tamarindusindica* et *Adansoniadigitata* (Kebenzikato et al. 2014; Padakale et al. 2015; Folega et al. 2019a; Atakpama et al. 2022a; Samarou et al. 2022)
- Ecological zone II: The northern mountain zone includes mountain vegetation formations in the north of the country, with dense dry forests containing *Anogeissusleiocarpa* and *Uapacatogoensis*, as well as open forests with *Isoberlinia* spp. (Dourma et al. 2012; Wala et al. 2012b; Atsri et al. 2018).
- Ecological zone III: The central plains zone, where vast areas are covered by dry forests with *Anogeissusleiocarpa*, as well as Guinean savannas dominated by Combretaceae and gallery forests along major waterways (Kokou et al. 2006).
- Ecological zone IV: The southern Togo mountain zone, where semi-deciduous forests characterize the region’s tropical Guinean mountainous climate, hosting diverse plant species like *Terminalia superba*, *Parinariglabra*, *Erythrophleumsuaveolens*, *Miliciaexcelsa*, *Antiarisafricana* et *Khayagrandifoliola*;
- Ecological zone V: The southern coastal zone, marked by highly degraded vegetation, forest remnants, gallery forests, mangroves, marshy wetlands, and grasslands (Batawila 1997).

Hydrographically, Togo is divided into three major basins: the Oti Basin (47.7%), the Mono Basin (37.7%), and the coastal basin of Lake Togo (14.6%). The country’s water resources primarily come from precipitation, which is drained by four main rivers (Oti, Mono, Haho, and Zio), with groundwater reserves estimated at over 9 billion cubic meters per year. Pedologically, the country has five main soil types classified into two categories: those with low agricultural potential (tropical ferruginous soils, poorly developed soils, vertisols, and hydromorphic soils) and those with high potential for good yields (ferrallitic soils) (Petit 1981).

### ﻿Data collection

Floristic data were collected from multiple sites representative of bioclimatic gradients (precipitation and latitude) in Togo between August–September 2022 and September–October 2023. These sites included 121 plots (Fig. 1B) randomly selected from a systematic grid of points spaced 5 km apart, generated using QGIS 3.24.1 and overlaid on the boundary layer of Togo. The study sites covered diverse areas, such as dense dry forests, open forests, savannas, as well as cultivated or fallow lands. The plot sizes were adapted to different vegetation types: 50 m × 20 m (Wouyo et al. 2023) for the aforementioned forested areas, 50 m × 10 m (Adjossou et al. 2022a) for riparian forests, and 50 m × 50 m (Banlipo et al. 2023) for agricultural fields and agroforestry parks, allowing the identification and naming of woody species. For herbaceous vegetation, a 10 m × 10 m subplot was established at each site.

Before data collection, a preliminary site assessment was conducted, and the plots were adjusted using high-resolution Google Earth Pro imagery. Species were recorded in each plot based on presence/absence. Species identification was carried out by referring to the analytical flora of Togo and Benin (Brunel et al. 1984; Akoègninou et al. 2006). The ecological conditions of each station were also documented, including a detailed description of the vegetation type, topography, and the presence of animals at each site.

### ﻿Data analysis

All field data collected were entered and processed in a Microsoft Excel spreadsheet. After data cleaning, the floristic analysis allowed for the establishment of a complete species list, following the APG IV phylogenetic nomenclature, available in the African Plant Database (https://africanplantdatabase.ch/en) (APGIV et al. 2016). The biological and phytogeographic types were determined following Raunkiaer (1934) and White (1986). The species’ conservation status was assessed using the IUCN Red List of Threatened Species, Version 2024-1 (https://www.iucnredlist.org). The invasive and/or alien nature of the species was evaluated based on multiple sources, including Akodéwou et al. (2019), who studied invasive species in southern Togo, Foxcroft et al. (2010), who analyzed invasive exotic flora in tropical and subtropical savannas, and the Global Invasive Species Database (UICN 2017). The list of vulnerable species in Togo was also considered (Radji and Akpene 2018). The identification of agroforestry and soil-fertility-enhancing species was based on various sources, including Samarou et al. (2023a) on cultivated and planted fertility-enhancing species, Atakpama et al. (2022b) on agroforestry systems, studies on agroforestry genetic resources (Ouro Djeri et al. 2001), and the World Agroforestry Centre (ICRAF) database (https://apps.worldagroforestry.org/treedb/), which lists species used in agroforestry systems worldwide. Statistical and descriptive analyses were performed using Microsoft Excel and R software (version 4.2.1). These analyses were used to calculate the percentage representation of botanical families, biological and phytogeographic types, assess the correlation of their frequencies, and generate various graphical representations to illustrate the results.

## ﻿Results

### ﻿General floristic overview

**Figure 2.**
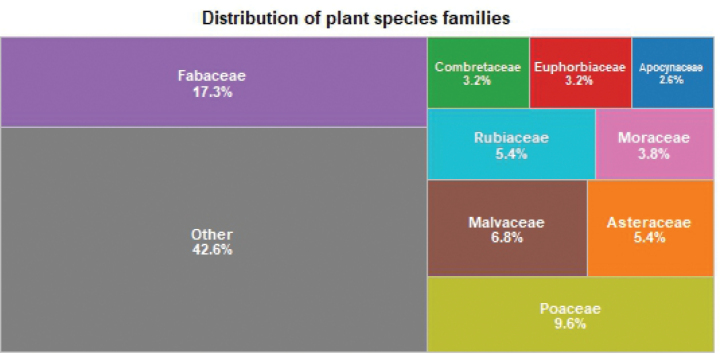
Treemap of plant species families. Treemap representing the relative species richness of the ten most common plant families recorded in the floristic inventory. Each rectangle’s size is proportional to the number of species in the family. The Fabaceae and Poaceae dominate, highlighting their ecological prevalence across Togo’s habitats.

The floristic exploration conducted in this study identified 498 plant species belonging to 92 families and 337 genera. Among these species, 86 are monocotyledons, 411 are dicotyledons, and one is a pteridophyte. Of the total recorded species, 69 are exotic, while 429 are native. The most diverse families are Fabaceae (86 species), Poaceae (48 species), Malvaceae (34 species), Rubiaceae (27 species), Asteraceae (27 species), Moraceae (19 species), Combretaceae (16 species), Euphorbiaceae (16 species), Apocynaceae (13 species), and Anacardiaceae (10 species) (Table 1) (Fig. 2).

**Table 1.** Distribution of plant species by predominant families.

Rank	Family	Number of species	Number of genera
**1**	**Fabaceae**	86	52
**2**	**Poaceae**	48	31
**3**	**Malvaceae**	34	15
**4**	**Rubiaceae**	27	22
**5**	**Asteraceae**	27	20
**6**	**Moraceae**	19	4
**7**	**Combretaceae**	16	4
**8**	**Euphorbiaceae**	16	13
**9**	**Apocynaceae**	13	12
**10**	**Anacardiaceae**	10	8

Based on species richness, the Fabaceae and Poaceae families stand out as the most diverse. Among the Fabaceae, the most represented species are *Piliostigmathonningii*, *Pterocarpuserinaceus* and *Danielliaoliveri*, while in the Poaceae family, *Andropogongayanus*, *Rottboelliaexaltata* and *Sporoboluspyramidalis* dominate.

**Figure 3.**
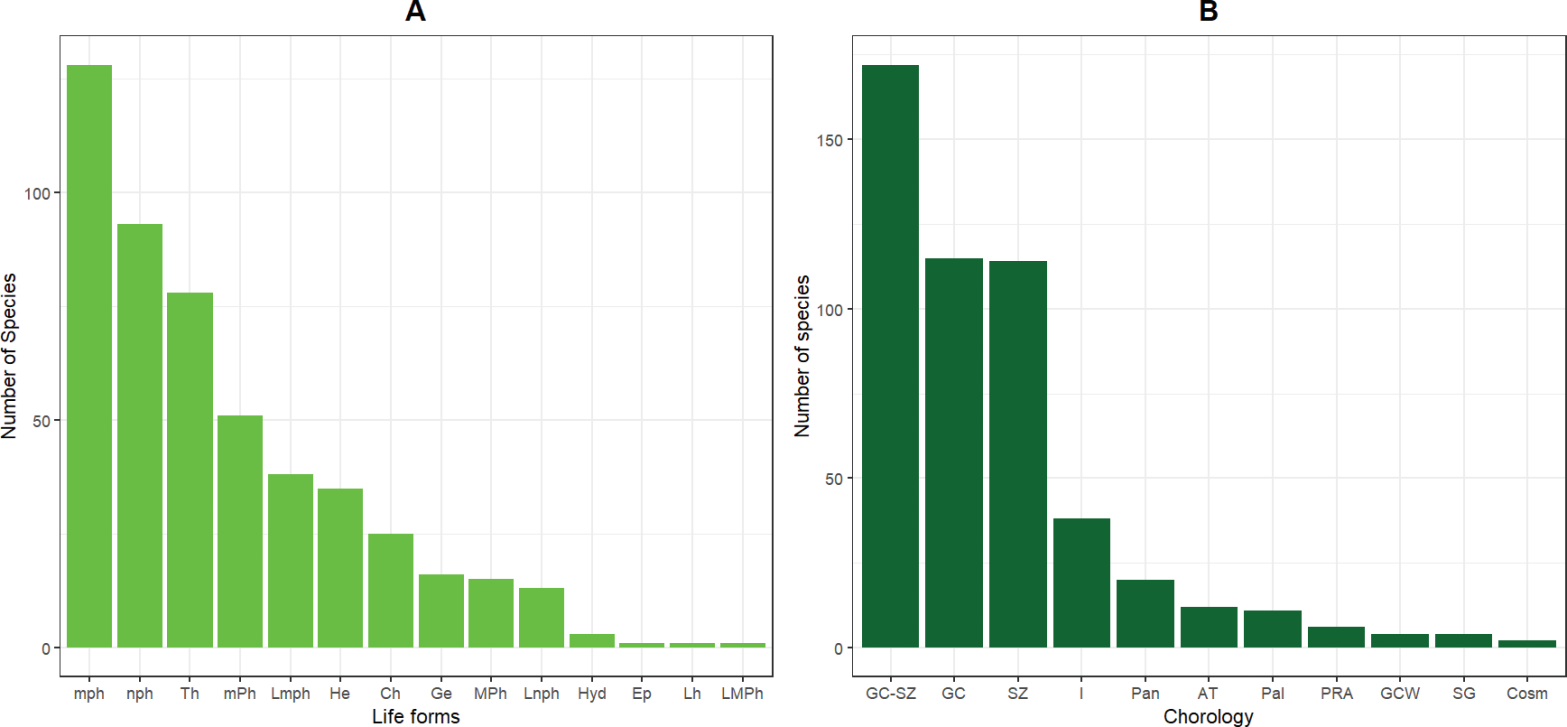
Distribution of life forms and chorological types of the recorded plant species. Legend: A. Bar chart showing the proportion of plant life forms: Ch: Chamaephytes, Ep: Epiphytes, Ge: Geophytes, He: Hemicryptophytes, Hyd: Hydrophytes, Lh: Liana Hemicryptophytes, Lnph: Liana Nanophanerophytes, Lmph: Liana Microphanerophytes, LMPh: Liana Megaphanerophytes, mph: Micophanerophytes, MPh: Megaphanerophytes, mPh: Mesophanerophytes, nph: Nanophanerophytes, Th: Therophytes; B. Chorological distribution illustrating species’ phytogeographical affinities: GC-SZ: Guinéo-Congolais/Soudano-Zambéziens, AT: Afrotropical, Cosm: Cosmopolitan, GC: Guineo-Congolese, GCW: Guinean Western, I: introduced, Pal: Paleotropical, Pan: Pantropical, SZ: Soudano-zambézian, SG: Soudan-Guinean.

The analysis of biological types reveals a predominance of phanerophytes, with a notable representation of microphanerophytes (25.7%) and nanophanerophytes (18.67%), followed by therophytes (15.06%). In contrast, some biological types are much less frequent, such as epiphytes, hemicryptophytic lianas, and megaphanerophytic lianas, each representing only 0.2% of the total (Fig. 3A). Regarding chorology, the majority of recorded species originate from the Guineo-Congolian/Sudano-Zambezian transition zone (34.54%), followed by the Guineo-Congolian (23.09%) and Sudano-Zambezian (29.89%) regions (Fig. 3B).

### ﻿Floristic diversity and ecological characteristics of the ecological zones

#### ﻿Ecological zone I

**Figure 4.**
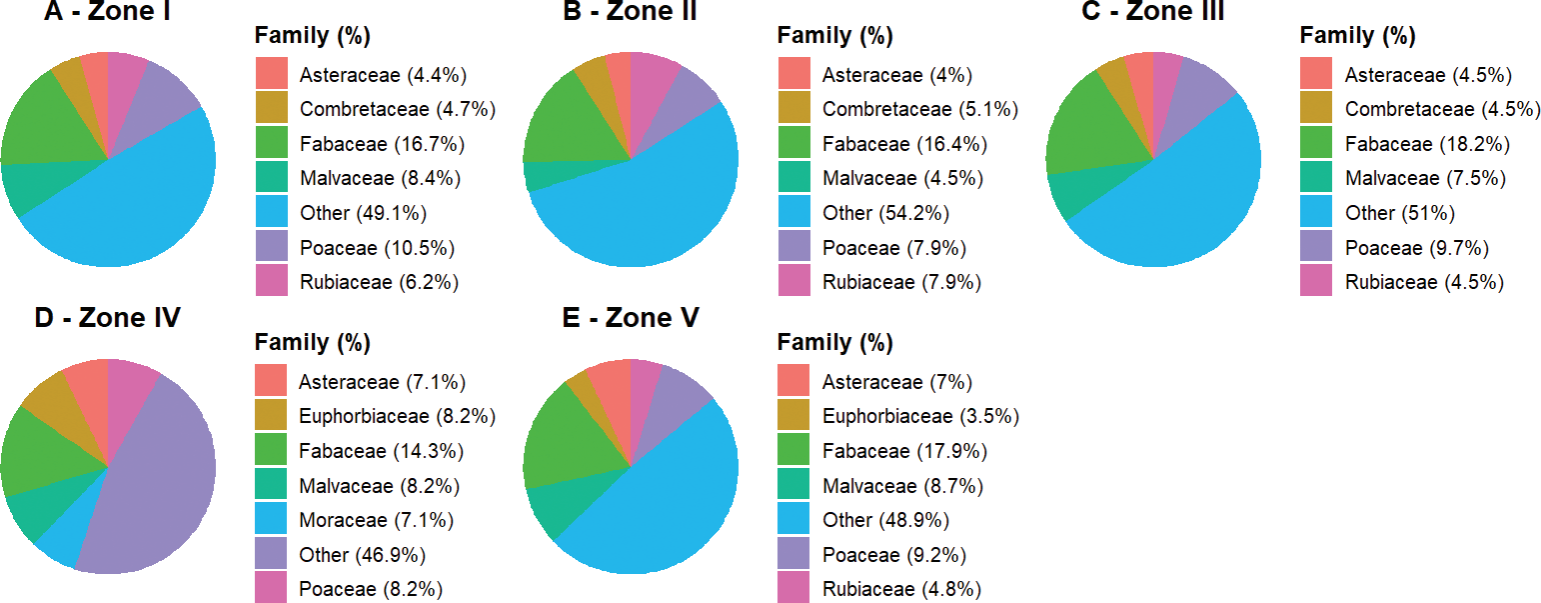
Distribution of plant families across different ecological zones. Stacked bar charts representing the relative abundance of dominant plant families within each ecological zone (I–V). The charts reveal spatial differences in species composition, with Fabaceae and Poaceae consistently dominant.

Ecological zone I, composed of fallows, agroforestry crops, and grassy savannas, exhibits a species diversity of 275 species distributed across 208 genera and 68 families, highlighting the zone’s significant floristic richness. The most represented families are Fabaceae (16.73%), Poaceae (10.55%), and Malvaceae (8.36%) (Fig. 4A), while genera such as Acacia (5 species), Combretum (6 species), and Ficus (5 species) dominate in terms of species count. Frequent species in this zone include *Parkiabiglobosa* and *Vitellariaparadoxa*. Regarding life forms, microphanerophytes (mp) (31.27%) and therophytes (Th) (16%) are the most common. Phytogeographically, most species are native (237 species), but a significant number of introduced (exotic) species (38 species) are also present, including *Azadirachtaindica*, *Leucaenaleucocephala*, and *Chromolaenaodorata*, which are invasive and/or alien species.

#### ﻿Ecological zone II

**Figure 5.**
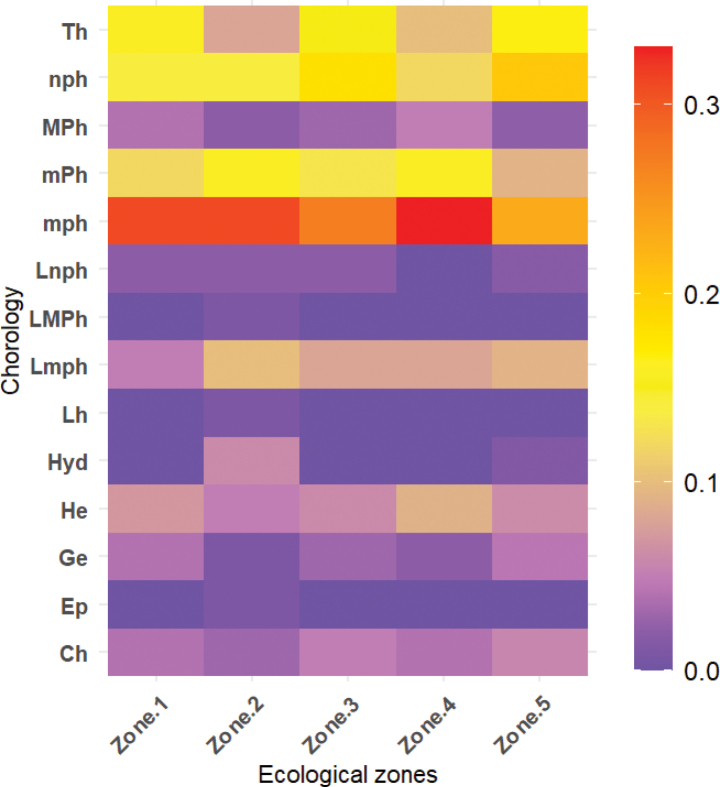
Distribution of biological types across the five ecological zones Heatmap visualizing the frequency of different plant life forms across ecological zones I–V.

Ecological zone II, characterized by dry dense forests, agroforestry crops, and savannas, contains 177 species across 142 genera and 54 families. The floristic diversity of this zone highlights the dominance of Fabaceae (29 species) and Poaceae (14 species) in terms of species richness (Fig. 4B). The most represented genera include *Andropogon* (4 species), *Dioscorea* (4 species), and *Ficus* (5 species), while *Annonasenegalensis* and *Ficussur* are frequent species. Life form analysis shows a dominance of phanerophytes, with high representation of microphanerophytes (30.51%) and mesophanerophytes (15.82%), followed by nanophanerophyte lianas (14.12%). Rare life forms, such as epiphytes and lianas, represent only 0.57% each (Fig. 5). Phytogeographically, most species originate from the Sudanian-Zambezian region (33.9%) and the Guineo-Congolian/Sudanian-Zambezian transition zone (31.07%). Introduced species (19 species) constitute a significant proportion, including *Chromolaenaodorata* and *Flueggeavirosa*, which are invasive and/or alien species.

#### ﻿Ecological zone III

Ecological zone III exhibits the highest species diversity, with 308 species across 220 genera and 65 families, showing a clear dominance of Fabaceae (56 species) and Poaceae (30 species) in terms of species richness (Fig. 4C). The most represented genera include *Ficus* (9 species), *Combretum* (6 species), and *Commelina* (5 species), while *Piliostigmathonningii*, *Vitellariaparadoxa*, and *Anogeissusleiocarpa* are predominant species. Phanerophytes dominate the life form distribution, with high representation of microphanerophytes (27.27%) and nanophanerophytes (17.86%), followed by therophytes (14.61%). Rare life forms include nanophanerophyte lianas (0.2%), megaphanerophytes (0.03%), and geophytes (0.3%). Phytogeographically, most species originate from the Guineo-Congolian/Sudanian-Zambezian transition zone (34.42%), the Guineo-Congolian region (22.4%), and the Sudanian-Zambezian region (21.75%). A total of 47 introduced species were recorded, including *Chromolaenaodorata* and *Calopogoniummucunoides*, both of which are invasive and/or alien species.

#### ﻿Ecological zone IV

Ecological zone IV, representing Togo’s forested zone, consists of mountainous and semi-deciduous dense forests. A total of 99 species, grouped into 87 genera and 32 families, were recorded. The dominant families are Fabaceae (14.3%), Poaceae (8.2%), Malvaceae (8.2%), Euphorbiaceae (8.2%), and Moraceae (7.1%) (Fig. 4D). The most represented genera include *Acacia*, *Ficus*, and *Combretum*, while *Antiarisafricana* is the dominant species. Phanerophytes dominate the life form spectrum, with high representation of microphanerophytes (32.65%), mesophanerophytes (16.32%), and nanophanerophytes (12.25%), followed by therophytes (10.2%). Less represented life forms include geophytes (0.02%), chamaephytes (0.04%), and megaphanerophytes (0.05%). Phytogeographically, most species originate from the Guineo-Congolian/Sudanian-Zambezian transition zone (34.54%), the Guineo-Congolian region (23.09%), and the Sudanian-Zambezian region (29.89%). Introduced (exotic) species (23 species) represent a notable proportion, including *Chromolaenaodorata*, *Azadirachtaindica*, and *Ageratumconyzoides*, which are invasive and/or alien species.

#### ﻿Ecological zone V

Ecological zone V, located along the coast, is primarily composed of wetlands and remnants of forests. The study revealed a species diversity of 229 plant species, grouped into 181 genera and 56 families, with Fabaceae dominating (17.9% of species), followed by Poaceae (9.17%) and Asteraceae (6.99%) (Fig. 4E), reflecting their ecological importance in the studied ecosystem. Life form analysis shows a predominance of phanerophytes, with a dominance of microphanerophytes (23.14%) and nanophanerophytes (20.52%). Phytogeographically, species from the Guineo-Congolian/Sudanian-Zambezian transition zone (GC-SZ: 39.74%), the Guineo-Congolian region (GC: 20.09%), and the Sudanian-Zambezian region (SZ: 19.21%) are highly represented. Introduced species account for 16.17% (24 species), primarily originating from tropical exogenous zones (Pan, Cosm), such as *Azadirachtaindica* and *Leucaenaleucocephala*. Many of these introduced species, including *Chromolaenaodorata* and *Pennisetumpolystachion*, are invasive and/or alien, posing a threat to local biodiversity due to their competitive nature.

### ﻿Biological and phytogeographical distributions across ecological zones

The heatmap of biological type frequencies across ecological zones highlights significant differences in the distribution of plant life forms (Fig. 5). Microphanerophytes exhibit the highest frequencies, particularly in Ecological zones I, II, and IV. Nanophanerophytes are also well represented in zones I and II. Ecological zone I stands out due to its high proportion of therophytes and nanophanerophytes. Mesophanerophytes are present in all ecological zones. Other biological types, such as lianas (Lnph, LMPh, Lh), epiphytes, geophytes, chamaephytes, hemicryptophytes, and megaphanerophytes, display much lower frequencies across the five ecological zones.

**Figure 6.**
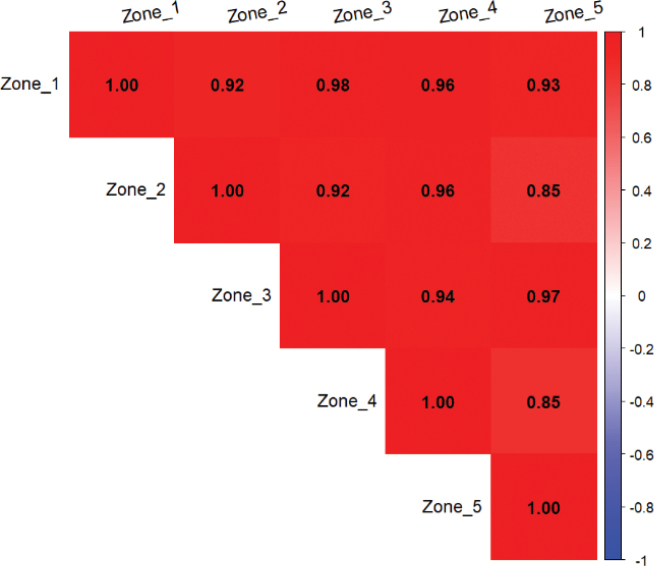
Correlation matrix of biological type frequencies across the five ecological zones Matrix showing Pearson correlation coefficients between the biological type distributions of different ecological zones.

The correlation matrix of the frequency of life forms across the five ecological zones shows high correlation values, ranging from 0.85 to 0.96, indicating a strong similarity in the distribution of life forms between these zones (Fig. 6). The strongest correlation is observed between zone I and zone III. In contrast, slightly weaker correlations are observed between zone II and zone I (0.85), and between zone IV and zone V (0.85).

**Figure 7.**
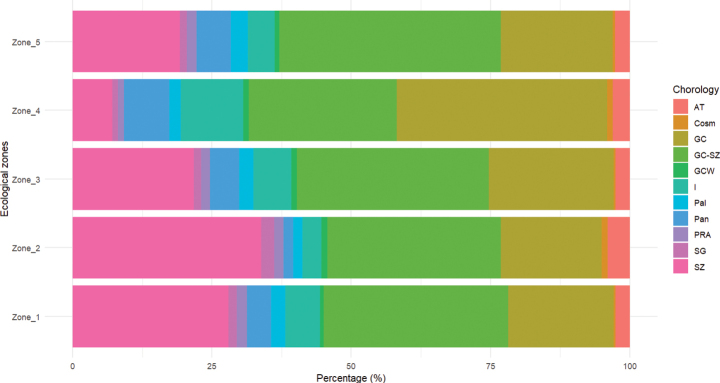
Phytogeographical distribution of the five ecological zones Stacked horizontal bar chart showing the percentage representation of phytogeographical types in each ecological zone.

The stacked horizontal bar chart illustrates the distribution of phytogeographical types across the five ecological zones (zone I to zone V) in percentage (Fig. 7). A strong predominance of GC (Guineo-Congolian) and GC-SZ (Guineo-Congolian-Sudanian-Zambezian), SZ (Sudano-Zambezian) types is observed in all zones, suggesting a strong influence of species from these biogeographical domains on the local flora. The types I (Introduced) and AT (Afro-tropical) are also well represented, although their proportion slightly varies between zones. Other phytogeographical types such as SG (Sudano-Guinean), PRA (Pluriregional African), Pan (Pantropical), Pal (Paleotropical), and GCW (Guinean Western) are minimally present in all zones.

**Figure 8.**
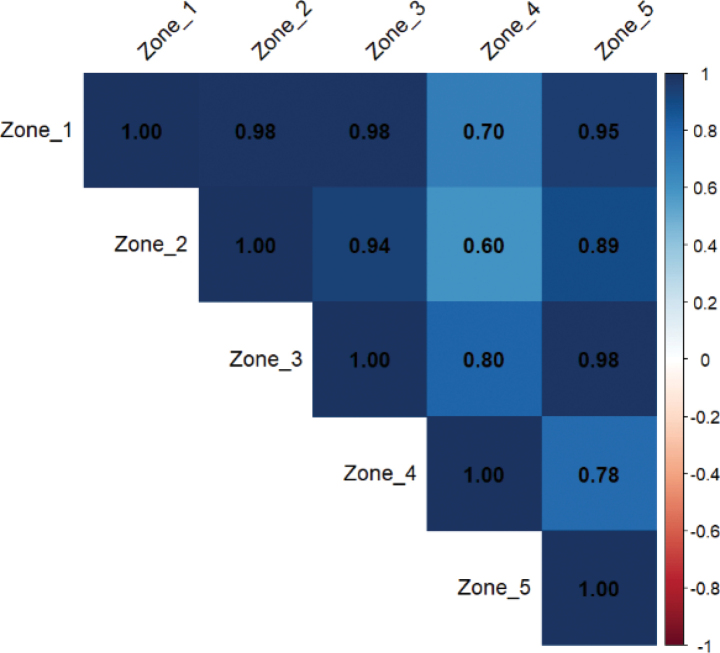
Chorological correlation of the five ecological zones. Correlation matrix of chorological frequencies among ecological zones. This figure highlights zones with similar floristic origins, supporting the interpretation of historical vegetation connectivity and environmental influences.

The correlation matrix of chorological frequency (Fig. 8) for the ecological zones highlights strong relationships between certain zones, suggesting similarities in floristic composition and environmental conditions. Strong correlations are observed between zone I and zone II (0.98).

### ﻿Threatened species, invasive and/or alien species, agroforestry species, and fertilizer species

According to the IUCN Red List, nine species are classified as near-threatened (NT), vulnerable (VU), or endangered (EN). These species are: *Miliciaexcelsa* (NT), *Albiziaferruginea* (NT), *Raphiasudanica* (NT), *Khayasenegalensis* (VU), *Khayagrandifoliola* (VU), *Garciniaafzelii* (VU), *Justiciaflava* (VU), *Tectonagrandis* (EN), and *Coffeaarabica* (EN). These geolocated species should be prioritized for conservation and protection to ensure their survival. A total of 48 harmful invasive and/or alien species have been recorded across all floras. All of these species are herbaceous, with *Chromolaenaodorata* (3.04%), *Tridaxprocumbens* (2.88%), and *Andropogongayanus* (2.8%) being the most dominant. These species represent a significant threat to local ecosystems due to their ability to spread rapidly and outcompete the species in the area. *Chromolaenaodorata*, *Commelinaerecta*, *Rottboelliaexaltata*, and *Tridax*procumbens are ubiquitous and found in all five ecological zones. Invasive/alien species are particularly abundant in Zone 1 (32 species), Zone 3 (36 species), and Zone 5 (32 species) (Table 2).

The recorded flora consists of 31 agroforestry species and 23 fertilizing species. Among the dominant agroforestry species, *Vitellariaparadoxa* (2.71%) and *Parkiabiglobosa* (2.04%) stand out, while the predominant fertilizing species is *Albiziazygia* (0.1%). Agroforestry species are particularly well-represented in ecological zones I and III, with 23 species each. In contrast, zones III and V have the highest number of fertilizing species, with 14 and 13 species, respectively (Table 2).

**Table 2.** distribution of invasive and/or alien species, agroforestry, and soil-fertility-enhancing species in the different ecological zones.

Ecological zone	Invasive and/or alien species	Agroforestry species	Soil-Fertility-Enhancing
**Zone I**	32	23	11
**Zone II**	19	14	6
**Zone III**	36	23	14
**Zone IV**	14	12	7
**Zone V**	32	15	13

## ﻿Discussion

### ﻿Floristic biodiversity and ecological factors

The number of Angiosperms recorded in this study is far from the species richness of Angiosperms reported in the flora of Togo. Although the flora list of Togo lacks regular updates, nearly 3,491 plant species were reported by Akpagana and Bouehet (1994). Since then, new discoveries and extinctions have not been considered to update this list. A literature review by par Folega et al. (2014b) noted a total of 3,468 species with 2,992 Angiosperms. A slight variation can be observed in these values. The variation between the two studies likely reflects a combination of factors related to research methods, scientific advancements, environmental changes, corrections of previous errors, extinctions, and new discoveries. The flora of 498 plant species in this study consists of 65 exotic species (introduced). This number is low and may be explained by the fact that collections were not made in urban areas, where a significant number of exotic species introduced for ornamental reasons can be found (Radji and Kokou 2015; Folega et al. 2019b).

The dominance of Fabaceae and Poaceae in this study is generally characterized by specific soil and climatic conditions, ecological dynamics (colonization, succession), and anthropogenic factors (agriculture, deforestation). It reflects both the resilience of these plants and the pressures exerted on ecosystems. This dominance may have positive implications (soil fertility, ecological resilience) but also highlights challenges (soil degradation, biodiversity loss). The two families occupy the largest proportions in the five ecological zones. According to Atakpama et al. (2019), the preponderance of Poaceae indicates the pyrophytic nature of the ecological zones of Togo.

The dominant presence of the Malvaceae, Rubiaceae, and Asteraceae families can be explained by several ecological and anthropogenic factors. These families particularly thrive in savannas and wetland environments, offering favorable conditions for their development. Akpamou et al. (2024) observed this strong dominance in southeastern Togo, contributing significantly to the floristic diversity of the Mono biosphere reserve. The notable presence of Rubiaceae is especially associated with riparian forests, an observation confirmed by Folega et al. (2014a) who documented a high proportion of Leguminosae-Papilionoideae (Fabaceae according to APG IV) and Rubiaceae in the riparian forests of the Sudanian zone of Togo. Furthermore, the presence of Combretaceae is characteristic of Sudanian endemism zones (Aubréville 1950), and their dominance in these zones has been highlighted by previous studies (Thiombiano et al. 2006; Folega et al. 2014b; Bawa et al. 2022). Thus, this family is well-represented in this study, particularly in ecological zones I and II. The presence of Moraceae and Euphorbiaceae could be related to biogeographical and anthropogenic factors. These families propagate favorably in humid forests, such as semi-deciduous forests and agroforests, which offer them adequate ecological conditions. In this study, their strong representation in ecological zones IV and V, which house forests and forest relics, confirms this trend. This result is consistent with previous work by Adjossou (2009) in the humid southern zone of Togo.

### ﻿Distribution and species diversity by ecological zones

The biological and phytogeographical types observed in the recorded plant formations reflect the diversity and distribution of plant communities, influenced by various environmental factors. This diversity reflects the ecological interactions and historical processes that shape the local flora. The dominance of phanerophytes (microphanerophytes, mesophanerophytes, and nanophanerophytes) in this study highlights the wooded nature of the plant formations encountered, particularly in ecological zones II and IV, where this dominance is especially pronounced. This observation is confirmed by the high correlation (0.96) of biological type frequencies in these two zones. Previous studies (Adjossou 2009; Atakpama et al. 2019) also emphasize the wooded nature of these ecological zones. The significant presence of Therophytes illustrates the xeric nature of certain environments. The highest proportion of Therophytes is observed in ecological zone I, characterized by environmental conditions marked by significant drought or limited water availability. In contrast, a hydrophyte species was occasionally observed, linked to the presence of waterholes in this zone. The highest proportion of hydrophytes is found in ecological zone V, which includes numerous flooded savannas and lakes.

Regarding phytogeographical types, there is a dominant presence of species belonging to the GC-SZ (Guineo-Congolese-Sudano-Zambesian), GC (Guineo-Congolese), and SZ (Sudano-Zambesian) categories. The strong dominance of Guineo-Congolese types in zone IV confirms the forested nature of this zone, while zone I stands out with a high proportion of Sudano-Zambesian types, highlighting the dry climatic conditions of the area. Other ecological zones show a significant proportion of transitional Guineo-Congolese-Sudano-Zambesian types. Zone III has the highest proportion of these transitional types, which may be explained by the presence of transitional species from the Atakora mountain range.

The distribution of species by ecological zones shows higher species richness in zone III. According to Ern (1979), ecological zones III and IV, which represent the Atakora mountain range, are the most diversified. This could be due to the mountainous topography of the area, which favors a microclimate conducive to high species richness and the presence of forests. Recent studies by Bilouktime et al. (2024) have also confirmed the high productivity of these ecological zones. Ecological zone II presents a lower diversity compared to Fazao-Malfakassa National Park, studied by Atakpama et al. (2023c), who recorded 26 species distributed across 126 genera and 101 families. Furthermore, Woegan et al. (2013) identified significant biodiversity in Fazao-Malfakassa National Park and the Alédjo Faunal Reserve, both located in this same ecological zone. In ecological zone IV, the species diversity recorded in this study is lower than that of Missahoé forest, where Atakpama et al. (2023b) recorded 241 plant species across 192 genera and 70 families. In contrast, the species richness of zone I is higher than that of Dough Fosse aux Lions, which includes 204 species distributed across 140 genera and belonging to 52 families (Atakpama et al. 2023a). The diversity of zone V exceeds that recorded by Fousséni et al. (2023) in the Togodo Faunal Reserve, where 201 species were recorded across 156 genera and 56 families. Finally, the species richness of ecological zone III is higher than that of Abdoulaye forest, studied by Wouyo et al. (2021), who recorded 162 plant species in 125 genera and 53 families. A comparison of the species diversity recorded in each ecological zone with the protected areas in these respective zones shows that some ecological zones, even without being strictly protected areas (since the inventories were not focused on these protected areas), can have high species richness. On the other hand, protected areas like Fazao-Malfakassa National Park and Missahoé forest stand out for their high diversity, confirming their essential role in plant species conservation. This suggests that effective management of protected areas is crucial for preserving biodiversity, and reinforced protection of certain ecological zones could further enhance conservation.

### ﻿Threats and conservation strategies

**Figure 9.**
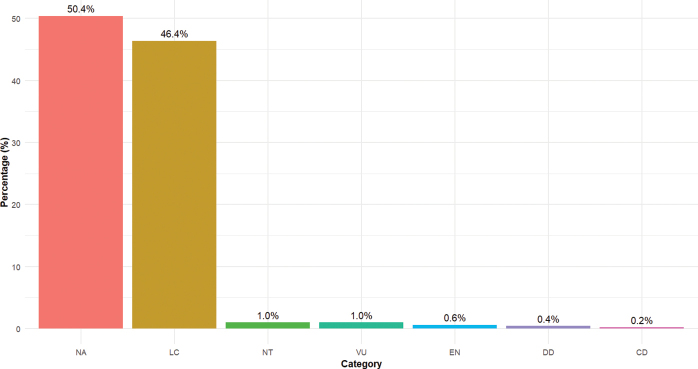
Red list category. Pie chart illustrating the conservation status of recorded species based on the IUCN Red List. Categories include Endangered VU: Vulnerable, EN: Endangered, LC: Least Concern, NT: Near threatened, NA: Not Assessed, DD: Data Deficient, CD: Conservation Dependent.

The flora recorded in this study reveals that 3.02% of plant species are threatened. The nine threatened species identified are primarily located in zone II, which houses Fazao-Malfakassa National Park, a key site for plant species conservation in the country. It is concerning to note that 50.4% of Togo’s plant species have not yet been assessed according to IUCN criteria, while 46.4% are classified as of least concern (Fig. 9). Similar results were obtained by Wouyo et al. (2023) in the Alibi-1 community forest.

These data highlight the urgency of strengthening conservation efforts, beginning with comprehensive studies to establish an exhaustive list of threatened species at the national level, in line with IUCN criteria. To date, such studies are scarce, and the few research efforts conducted, notably by Radji and Akpene (2018), represent one of the only references on threatened plants in Togo. Additionally, a study identified 137 threatened minor food species, underscoring the need for specific conservation measures for these plants (Akpavi et al. 2012). Rapid deforestation, mainly caused by extensive agriculture, logging, bushfires, and the high demand for wood fuel, poses a major threat to Togo’s flora (Badjare et al. 2021). Today, it is becoming difficult to find species that are not under threat due to these anthropogenic pressures. In contrast, the proliferation of invasive and/or aggressive species, whose list has been established in this study, is observed. This phenomenon results from anthropogenic pressures on the flora, as widely demonstrated by Akodéwou et al. (2019) on invasive plants in southern Togo and by Atakpama et al. (2019) on pyrophytic plants.

The protection of threatened plant species in Togo requires strengthening the legal and institutional framework, more effective management of protected areas, and the promotion of agroforestry and sustainable agricultural practices. It is essential to conduct in-depth studies on biodiversity to better understand the status of threatened species, their habitats, and the threats they face. Furthermore, raising awareness among local communities, integrating conservation into education, and developing scientific research, particularly through ex-situ conservation and ecosystem restoration, are crucial. Conservation efforts in Togo must contend with significant socioeconomic barriers, particularly rural poverty. Rural dependence on wood fuel and subsistence agriculture often undermines conservation goals (Newmark and Hough 2000). Integrating community-based resource management, sustainable agroforestry, and conservation incentives could help bridge ecological and human development priorities. Finally, national and international cooperation and mobilization of funding are essential to ensure the effective and sustainable conservation of Togo’s biodiversity.

Regarding agroforestry and fertility species, their integration into Togo’s agricultural practices could not only improve soil fertility but also promote the diversification of agricultural production and strengthen the resilience of systems to climate hazards. However, the available research documenting and analyzing these species remains relatively limited. Some studies have identified fruit species in agroforestry systems in the Plateaux region of Togo (Atakpama et al. 2022b; Samarou et al. 2023a), and the list established in this study enriches this knowledge. Other sporadic studies (Padakale et al. 2015; Atakpama et al. 2022a; Samarou et al. 2023b) have been conducted on agroforestry parks, fitting into the emerging dynamic of organic farming and the rethinking of intensive production based on chemical fertilizers. It is essential that these in-depth studies on agroforestry and fertility species continue to better understand the multiple functions of agroforestry species and fully leverage them for the sustainable development of the agroforestry and agricultural sectors in Togo.

## ﻿Conclusion

The findings of this study underscore the critical importance of regularly updating floristic data to enhance our understanding of ecological dynamics and effectively inform conservation strategies. The diverse ecological zones of Togo, particularly zone III, exhibit significant floristic richness, highlighting the need for targeted surveys and assessments. The inventory revealed 498 plant species, including nine classified as threatened according to IUCN criteria, while more than 50% of the species remain unassessed, indicating a substantial gap in knowledge that must be addressed. The study also documents a concerning prevalence of invasive species, which, coupled with rapid anthropogenic pressures such as deforestation and climate change, poses a severe risk to native biodiversity. An integrated approach that combines community engagement, strengthened institutional frameworks, and international cooperation is essential to safeguard Togo’s unique plant heritage. This research serves as a foundation for future studies and provides crucial insights necessary for the implementation of effective conservation measures. Continued exploration and floristic assessment remain priorities to ensure the sustainable management of Togo’s natural resources and to protect its ecological integrity in the face of ongoing environmental challenges. It is also important to emphasize the opportunity that this publication represents to draw the attention of decision-makers to the urgent need to update the 1984 Flora of Togo, in order to have an up-to-date scientific reference that is adapted to contemporary ecological realities.

## ﻿Additional information

### Use of AI

No use of AI was reported.

### Author ORCIDs

Folega Fousséni https://orcid.org/0000-0001-9097-3524

Bawa Demirel Maza-esso https://orcid.org/0000-0003-4823-647X

Atakpama Wouyo https://orcid.org/0000-0001-7041-918X

Badjaré Bilouktime https://orcid.org/0009-0006-8139-433X

Jasmina Šinžar-Sekulić https://orcid.org/0000-0002-8181-7164

Wala Kperkouma https://orcid.org/0000-0002-7533-6356

Batawila Komlan https://orcid.org/0000-0003-2781-3063

Akpagana Koffi https://orcid.org/0000-0003-4290-8861

### Data availability

The dataset associated with this study has been deposited in an external repository and is publicly accessible. The dataset can be accessed at the Zenodo repository via the following link: https://zenodo.org/records/14889506.

## Supplementary materials

Supplementary material 1 Baseline data of the recorded species Data type docx https://binary.pensoft.net/file/1396838This dataset is made available under the Open Database License (http://opendatacommons.org/licenses/odbl/1.0/). The Open Database License (ODbL) is a license agreement intended to allow users to freely share, modify, and use this Dataset while maintaining this same freedom for others, provided that the original source and author(s) are credited.Folega Fousséni, Bawa Demirel Maza-esso, Atakpama Wouyo, Badjaré Bilouktime, Jasmina Šinžar-Sekulić, Wala Kperkouma, Batawila Komlan, Akpagana Koffi
