# Supplementary material for: ﻿Floristic diversity and assessment of the conservation status of Togo’s plant species
Source: PhytoKeys. 2025 Aug 19;261:211–32. doi: 10.3897/phytokeys.261.151951 (PMC12381583; doi:10.3897/phytokeys.261.151951)
Supplement: Supplementary material 1 — Baseline data of the recorded species [file phytokeys-261-211_article-151951__-s001.docx]

**Supplementary Data**

**Appendix A: List of Recorded Species**

**Life Forms**: Ch: Chamaephytes, Ep: Epiphytes, Gr: Geophytes, He: Hemicryptophytes, Hyd: Hydrophytes, Lge: Liana Geophytes, LH: Liana Hemicryptophytes, Lnp: Liana Nanophanerophytes, Lmp: Liana Microphanerophytes, LMP: Liana Megaphanerophytes, mp: Micophanerophytes, MP: Megaphanerophytes, Mp: Mesophanerophytes, np: Nanophanerophytes, Th: Therophytes

**Chorology**: GC-SZ : Guinéo-Congolais/Soudano-Zambéziens, AT: Afrotropical, Cosm: Cosmopolitan, GC: Guineo-Congolese, GCW: Guinean Western, I: introduced, Pal: Paleotropical, Pan: Pantropical, SZ: Soudano-zambézian, SG: Soudan-Guinean

VU: Vulnerable, EN: Endangered, LC: Least Concern, NT: Near threatened, NA: Not Assessed, DD: Data Deficient, CD: Conservation Dependent

D: Dicotyledon, M: Monocotyledon, P: Pteridophyt

| N° | Species | Family | TB | TP | CLASS | Status | Origin |
| --- | --- | --- | --- | --- | --- | --- | --- |
| 1 | *Abrus precatorius* **L. subsp. africanus Verdc.** | Fabaceae | Lmph | GC-SZ | D | NA | Native |
| 2 | *Abutilon mauritianum* **(Jacq.) Medic.** | Malvaceae | nph | GC-SZ | D | NA | Native |
| 3 | *Acacia ataxacantha* **DC.** | Fabaceae | mph | GC-SZ | D | LC | Native |
| 4 | *Acacia auriculiformis* **A. Cunn. ex Benth.** | Fabaceae | mph | I | D | LC | Exotic |
| 5 | *Acacia gourmaensis* **A Chev.** | Fabaceae | mph | SZ | D | NA | Native |
| 6 | *Acacia macrostachya* **Reichenb.** | Fabaceae | mph | SZ | D | LC | Native |
| 7 | *Acacia nilotica* **(L.) Willd.** | Fabaceae | mph | SZ | D | LC | Exotic |
| 8 | *Acacia polyacantha* **Willd.** | Fabaceae | mPh | GC-SZ | D | NA | Native |
| 9 | *Acacia seyal* **DC.** | Fabaceae | mph | SZ | D | LC | Native |
| 10 | *Acacia sieberiana* **DC.** | Fabaceae | mph | SZ | D | NA | Native |
| 11 | *Acalypha ciliata* **Forssk.** | Euphorbiaceae | Th | GC | D | NA | Native |
| 12 | *Acanthospermum hispidum* **DC.** | Asteraceae | Th | GC-SZ | D | NA | Exotic |
| 13 | *Acanthus montanus* **(Nees) T.** **Anders.** | Acanthaceae | nph | Pan | D | LC | Native |
| 14 | *Acroceras amplectens* **Stapf** | Poaceae | nph | SZ | M | LC | Native |
| 15 | *Adansonia digitata* **L.** | Malvaceae | MPh | SZ | D | NA | Native |
| 16 | *Adenodolichos paniculatus* **(Hua) Hutch. & Dalz.** | Fabaceae | Ch | SZ | D | NA | Native |
| 17 | *Aeschynomene sensitiva* **Sw.** | Fabaceae | nph | GC-SZ | D | LC | Native |
| 18 | *Aframomum alboviolaceum* **(Ridl.) K. Schum.** | Zingiberaceae | nph | GC | D | LC | Native |
| 19 | *Aframomum sceptrum* **(Oliv. & Hanb.) K Schum.** | Zingiberaceae | nph | GC | D | LC | Native |
| 20 | *Aframomum tectorum* **K. Schum***.* | Zingiberaceae | nph | GC | D | LC | Native |
| 21 | *Afzelia africana* **Sm.** | Fabaceae | mPh | SZ | D | NA | Native |
| 22 | *Ageratum conyzoides* **L.** | Asteraceae | Th | GC-SZ | D | LC | Exotic |
| 23 | *Albizia adianthifolia* ***(*Schumach.) W.Wight** | Fabaceae | mph | GC | D | LC | Native |
| 24 | *Albizia chevalieri* **Harms** | Fabaceae | mph | GC | D | NA | Native |
| 25 | *Albizia ferruginea* **(Guill. & Perr.) Benth.** | Fabaceae | mph | GC | D | NT | Native |
| 26 | *Albizia lebbeck* **(L.) Benth.** | Fabaceae | mph | I | D | LC | Exotic |
| 27 | *Albizia zygia* **(DC.) J.F. Macbr.** | Fabaceae | mph | GC | D | LC | Native |
| 28 | *Alchornea cordifolia* **(Schumach. & Thonn.) Müll.Arg.** | Euphorbiaceae | Lmph | AT | D | LC | Native |
| 29 | *Allophylus africanus* **P.Beauv.** | Sapindaceae | mph | GC | D | LC | Native |
| 30 | *Allophylus cobbe* **(L.) Reausch.** | Sapindaceae | mph | GC-SZ | D | NA | Native |
| 31 | *Aloe vera* **(L.) Burm.f.** | Asphodelaceae | He | I | M | NA | Exotic |
| 32 | *Alternanthera brasiliana* **(L.) Kuntze** | Amaranthaceae | Ch | I | D | NA | Exotic |
| 33 | *Alysicarpus ovalifolius* **(Schum. & Thonn.) J. Léonard** | Fabaceae | Th | GC-SZ | D | NA | Native |
| 34 | *Alysicarpus vaginalis* **(L.) DC.** | Fabaceae | Th | GC-SZ | D | NA | Native |
| 35 | *Alysicarpus zeyheri* **Harv.** | Fabaceae | Th | GC-SZ | D | NA | Native |
| 36 | *Amorphophallus dracontioides* **(Engl.) N.E.Br.** | Araceae | Lmph | AT | M | NA | Native |
| 37 | *Ampelocissus bombycina* **(Baker) Planch.** | Vitaceae | Lmph | GC-SZ | D | NA | Native |
| 38 | *Ampelocissus multistriata* **(Bak.) Planch.** | Vitaceae | Lmph | GC-SZ | D | NA | Native |
| 39 | *Anacardium occidentale* **L.** | Anacardiaceae | mph | I | D | LC | Exotic |
| 40 | *Ananas comosus* **( L. ) Merrill** | Bromeliaceae | He | I | M | NA | Exotic |
| 41 | *Anchomanes difformis* **(Bl.) Engl.** | Araceae | Ge | GC | M | LC | Native |
| 42 | *Andropogon africanus* **Franch.** | Poaceae | He | GC-SZ | M | LC | Native |
| 43 | *Andropogon chinensis* **(Nees) Merr.** | Poaceae | He | GC-SZ | M | NA | Native |
| 44 | *Andropogon contortus* **L.** | Poaceae | He | GC-SZ | M | NA | Native |
| 45 | *Andropogon gayanus* **kunth var. gayanus** | Poaceae | He | GC-SZ | M | NA | Native |
| 46 | *Andropogon tectorum* **Schum. & Thonn.** | Poaceae | He | GC-SZ | M | NA | Native |
| 47 | *Aneilema lanceolatum* **Benth.** | Commelinaceae | Ch | GC | M | NA | Native |
| 48 | *Aneilema paludosum* **A.Chev. subsp. Paludosum** | Commelinaceae | Ch | GC | M | NA | Native |
| 49 | *Annona senegalensis* **Pers.** | Annonaceae | nph | GC-SZ | D | LC | Native |
| 50 | *Anogeissus leiocarpa* **(DC.) Guill. & Perr.** | Combretaceae | mPh | SZ | D | LC | Native |
| 51 | *Anthocleista nobilis* **G.Don** | Gentaniaceae | mPh | GC | D | LC | Native |
| 52 | *Antiaris africana* **Engl.** | Moraceae | MPh | GC | D | NA | Native |
| 53 | *Antiaris toxicaria* **Lesch.** | Moraceae | mPh | GC-SZ | D | LC | Native |
| 54 | *Antidesma venosum* **E.Mey.** | Euphorbiaceae | nph | GC-SZ | D | NA | Native |
| 55 | *Arachis hypogaea* **L.** | Fabaceae | Th | I | D | NA | Exotic |
| 56 | *Aristida adscensionis* **L.** | Poaceae | Th | GC-SZ | M | NA | Native |
| 57 | *Asparagus flagellaris* **(Kunth) Bak.** | Asparagaceae | Lnph | GC-SZ | D | NA | Native |
| 58 | *Aspilia bussei* **O.Hoffm. & Muschl.** | Asteraceae | nph | SZ | D | NA | Native |
| 59 | *Aspilia helianthoides* **(Schumach. & Thonn.) Oliv. & Hiern** | Asteraceae | nph | GC-SZ | D | LC | Native |
| 60 | *Asystasia gangetica* **(L.) T.Anderson** | Acanthaceae | nph | Pan | D | NA | Native |
| 61 | *Aubrevillea kerstingii* **(Harms) Pellegr.** | Fabaceae | MPh | GC | D | LC | Native |
| 62 | *Azadirachta indica* **A. Juss.** | Meliaceae | mph | I | D | LC | Exotic |
| 63 | *Balanites aegyptiaca* **(L.) Delile** | Zygophyllaceae | mph | SZ | D | LC | Native |
| 64 | *Bambusa vulgaris* **Schrad.** | Poaceae | He | I | M | NA | Exotic |
| 65 | *Berlinia grandiflora* **(Vahl) Hutch. & Dalziel** | Fabaceae | mPh | GC-SZ | D | LC | Native |
| 66 | *Bidens* **pilosa L.** | Asteraceae | Th | GC-SZ | D | NA | Native |
| 67 | *Biophytum petersianum* **Klotzch** | Oxalidaceae | Th | Pal | D | NA | Native |
| 68 | *Blighia sapida* **K.D.Koenig** | Sapindaceae | mPh | Pan | D | LC | Exotic |
| 69 | *Boerhavia erecta L.* | Nyctaginaceae | Th | Pan | D | NA | Native |
| 70 | *Bombax costatum* **Pellegr. & Vuillet** | Malvaceae | mph | SZ | D | LC | Native |
| 71 | *Borassus aethiopum* **Mart.** | Arecaceae | MPh | SZ | M | LC | Native |
| 72 | *Boswellia dalzielii* **Hutch.** | Burseraceae | mph | SZ | D | NA | Native |
| 73 | *Brachiaria deflexa* **(Schumach.) C.E.Hubb. ex Robyns** | Poaceae | Th | GC-SZ | M | NA | Native |
| 74 | *Brachiaria lata* **(Schumach.) C.E.Hubb.** | Poaceae | Th | GC-SZ | M | NA | Native |
| 75 | *Bridelia ferruginea* **Benth.** | Phyllantaceae | nph | GC-SZ | D | LC | Native |
| 76 | *Bridelia micrantha* **(Hochst.) Baill.** | Phyllantaceae | nph | GC-SZ | D | LC | Native |
| 77 | *Burkea africana* **Hook.** | Fabaceae | mph | SZ | D | LC | Native |
| 78 | *Caesalpinia bonduc* **(L.) Roxb.** | Fabaceae | Lmph | GC | D | LC | Native |
| 79 | *Cajanus cajan* **(L.) Millsp.** | Fabaceae | nph | I | D | NT | Exotic |
| 80 | *Calopogonium mucunoides* **Desv.** | Fabaceae | Lmph | GC | D | NA | Exotic |
| 81 | *Calotropis procera* **(Aiton) W.T. Aiton** | Apocynaceae | mph | GC | D | LC | Exotic |
| 82 | *Calyptrochilum christyanum* **(Rchb. f.) Summerh.** | Orchidaceae | Ep | GC-SZ | M | NA | Native |
| 83 | *Canarium schweinfurthii* **Engl.** | Burseraceae | MPh | GC | D | LC | Native |
| 84 | *Canthium venosum* **(Oliv.) Hiern** | Rubiaceae | Lmph | GC | D | NA | Native |
| 85 | *Capsicum annuum* **L.** | Solanaceae | nph | I | D | LC | Exotic |
| 86 | *Caralluma diffusa* **(Wight) N.E.Br.** | Apocynaceae | nph | SZ | D | NA | Native |
| 87 | *Carissa edulis* **Vahl** | Apocynaceae | Lmph | SZ | D | LC | Native |
| 88 | *Carissa spinarum* **L.** | Apocynaceae | mph | Pal | D | LC | Native |
| 89 | *Ceiba pentandra* **(L.) Gaertn.** | Malvaceae | MPh | Pan | D | LC | Native |
| 90 | *Celosia trigyna* **L.** | Amaranthaceae | Th | GC-SZ | D | NA | Native |
| 91 | *Celtis mildbraedii* **Engl.** | Ulmaceae | MPh | GC | D | LC | Native |
| 92 | *Centrosema pubescens* **Benth.** | Fabaceae | Lmph | GC | D | NA | Exotic |
| 93 | *Ceratotheca sesamoides* **Endl.** | Pedaliaceae | He | SZ | D | NA | Native |
| 94 | *Chamaecrista mimosoides* **(L.) Greene** | Fabaceae | nph | SZ | D | LC | Native |
| 95 | *Chassalia kolly* **(Schumach.) Hepper** | Rubiaceae | nph | GCW | D | NA | Native |
| 96 | *Chloris pilosa* **Schumach.** | Poaceae | Th | GC-SZ | M | NA | Native |
| 97 | *Chromolaena odorata* **(L.) R.M.King & H.Rob.** | Asteraceae | nph | GC | D | NA | Exotic |
| 98 | *Cissampelos mucronata* **A.Rich.** | Menispermaceae | Lmph | GC-SZ | D | NA | Native |
| 99 | *Cissus aralioides* **Welw. ex Bak.** | Vitaceae | Lmph | GC-SZ | D | NA | Native |
| 100 | *Cissus petiolata* **Hook.f.** | Vitaceae | Lmph | GC | D | NA | Native |
| 101 | *Cissus populnea* **Guill. & Perr** | Vitaceae | Lmph | AT | D | NA | Native |
| 102 | *Cissus quadrangularis* **L.** | Vitaceae | Lnph | SZ | D | NA | Native |
| 103 | *Citrus limon* **(L.) Burm. f.** | Rutaceae | mph | I | D | LC | Exotic |
| 104 | *Citrus sinensis* **(L.)** | Rutaceae | mph | I | D | NA | Exotic |
| 105 | *Cleistopholis patens* **(G. Bentham) H.G. Engler & F.L. Diels** | Annonaceae | mPh | GC | D | LC | Native |
| 106 | *Clematis hirsuta* **Perr. & Guill.** | Ranunculaceae | Lh | SZ | D | NA | Native |
| 107 | *Cleome viscosa* **L.** | Cleomaceae | Th | GC-SZ | D | NA | Native |
| 108 | *Clerodendrum capitatum* **(Willd.) Schumach.** | Verbenaceae | nph | GC-SZ | D | LC | Native |
| 109 | *Clitoria falcata* **Lam.** | Fabaceae | Lnph | GC-SZ | D | NA | Native |
| 110 | *Cnestis ferruginea* **DC.** | Connaraceae | Lmph | GC | D | NA | Native |
| 111 | *Cochlospermum planchonii* **Hook. f. ex Planch.** | Cochlospermaceae | nph | SZ | D | NA | Native |
| 112 | *Coffea arabica* **L.** | Rubiaceae | mph | I | D | EN | Exotic |
| 113 | *Coffea canephora* **Pierre ex Froehner** | Rubiaceae | nph | I | D | LC | Exotic |
| 114 | *Cola gigantea* **A.Chev.** | Malvaceae | mPh | GC | D | LC | Native |
| 115 | *Cola millenii* **K. Schum.** | Malvaceae | mph | GC | D | LC | Native |
| 116 | *Cola nitida* **(Vent.) Scott & Endl.** | Malvaceae | mPh | GC | D | LC | Native |
| 117 | *Combretum glutinosum* **Perr.** | Combretaceae | mph | SZ | D | LC | Native |
| 118 | *Combretum mucronatum* **Schum.** | Combretaceae | mph | SZ | D | NA | Native |
| 119 | *Combretum collinum* **Fresen.** | Combretaceae | mph | GC | D | LC | Native |
| 120 | *Combretum fragrans* **F. Hoffm.** | Combretaceae | mph | SZ | D | NA | Native |
| 121 | *Combretum molle* **G. Don** | Combretaceae | mph | SZ | D | LC | Native |
| 122 | *Combretum nigricans* **Lepr.** | Combretaceae | mph | SZ | D | LC | Native |
| 123 | *Combretum paniculatum* **Vent.** | Combretaceae | Lmph | SZ | D | NA | Native |
| 124 | *Combretum sericeum* **G. Don** | Combretaceae | He | SZ | D | NA | Native |
| 125 | *Commelina africana* **L.** | Commelinaceae | Ch | GCW | M | LC | Native |
| 126 | *Commelina aspera* **Benth.** | Commelinaceae | Ch | SZ | M | NA | Native |
| 127 | *Commelina benghalensis* **L.** | Commelinaceae | Ch | Pal | M | LC | Exotic |
| 128 | *Commelina diffusa* **Burm.f.** | Commelinaceae | Ch | Pan | M | LC | Native |
| 129 | *Commelina erecta* **L. subsp. Erecta** | Commelinaceae | Ch | Cosm | M | LC | Native |
| 130 | *Commelina forskalaei* **Vahl** | Commelinaceae | Ch | Pal | M | NA | Native |
| 131 | *Corchorus aestuans* **L.** | Malvaceae | Th | GC-SZ | D | NA | Native |
| 132 | *Corchorus olitorius* **L.** | Malvaceae | Th | GC-SZ | D | NA | Native |
| 133 | *Corchorus tridens* **L.** | Malvaceae | Th | GC-SZ | D | NA | Native |
| 134 | *Cordia guineensis* **Schum. & Thonn.** | Boraginaceae | nph | GC-SZ | D | LC | Native |
| 135 | *Cordia africana* **Lam.** | Boraginaceae | nph | GC | D | LC | Native |
| 136 | *Costus afer* **Ker–Gawl.** | Zingiberaceae | nph | GC | D | NA | Native |
| 137 | *Crassocephalum rubens* **(Juss. ex Jacq.) Moore** | Asteraceae | Th | SZ | D | NA | Native |
| 138 | *Crinum jagus* **(J.Thomps.) Dandy** | Amaryllidaceae | Ge | GC-SZ | M | LC | Native |
| 139 | *Crossopteryx febrifuga* **(Afzel.) Benth.** | Rubiaceae | mph | SZ | D | LC | Native |
| 140 | *Crotalaria juncea* **L.** | Fabaceae | Th | GC-SZ | D | NA | Native |
| 141 | *Crotalaria lanceolata* **E.Mey.** | Fabaceae | nph | GC-SZ | D | LC | Native |
| 142 | *Crotalaria naragutensis* **Hutch.** | Fabaceae | Th | SZ | D | NA | Native |
| 143 | *Crotalaria pallida* **Ait. var. pallida** | Fabaceae | nph | GC-SZ | D | NA | Native |
| 144 | *Crotalaria retusa* **L.** | Fabaceae | Ch | SZ | D | NA | Native |
| 145 | *Croton lobatus* **L.** | Euphorbiaceae | Th | GC-SZ | D | NA | Native |
| 146 | *Curculigo pilosa* **(Schumach. & Thonn.) Engl.** | Hypoxidaceae | He | SZ | D | NA | Native |
| 147 | *Cussonia arborea* **Hoehst.** | Araliaceae | mph | SZ | D | LC | Native |
| 148 | *Cymbopogon giganteus* **Chiov.** | Poaceae | He | SZ | M | NA | Native |
| 149 | *Cynodon dactylon* **(L.) Pers.** | Poaceae | Ch | GC-SZ | M | NA | Native |
| 150 | *Cynometra megalophylla* **Harms** | Fabaceae | mPh | GC | D | LC | Native |
| 151 | *Cyperus alopecuroides* **Rottb.** | Cyperaceae | Ge | SZ | M | LC | Native |
| 152 | *Cyperus amabilis* **Vahl** | Cyperaceae | Th | GC-SZ | M | LC | Native |
| 153 | *Cyperus articulatus* **L.** | Cyperaceae | Hyd | GC-SZ | M | LC | Native |
| 154 | *Cyperus pectinatus* **Vahl** | Cyperaceae | He | GC-SZ | M | LC | Native |
| 155 | *Cyperus rotundus* **L.** | Cyperaceae | Ge | GC-SZ | M | NA | Exotic |
| 156 | *Cyphostemma adenocaule* **(Steud.) Desc.** | Vitaceae | Lmph | GC-SZ | D | NA | Native |
| 157 | *Dactyloctenium aegyptium* **(L.) Willd.** | Poaceae | Ch | Pal | M | NA | Exotic |
| 158 | *Daniellia oliveri* **(Rolfe) Hutch. & Dalziel** | Fabaceae | mPh | SZ | D | LC | Native |
| 159 | *Desmodium ramosissimum* **G. Don** | Fabaceae | Ch | GC-SZ | D | NA | Native |
| 160 | *Desmodium adscendens* **(Sw.) DC var. adscendens** | Fabaceae | Ch | GC | D | LC | Exotic |
| 161 | *Desmodium gangeticum* **(L.) DC. var. gangeticum** | Fabaceae | Ch | GC-SZ | D | NA | Native |
| 162 | *Desmodium tortuosum* **(Sw.) DC.** | Fabaceae | nph | GC-SZ | D | NA | Native |
| 163 | *Desmodium velutinum* **(Willd.) DC.** | Fabaceae | nph | GC-SZ | D | NA | Native |
| 164 | *Detarium microcarpum* **Guill. & Perr.** | Fabaceae | mph | SZ | D | LC | Native |
| 165 | *Dialium guineense* **Willd.** | Fabaceae | mPh | GC | D | LC | Native |
| 166 | *Dichapetalum madagascariense* **Poir.** | Dichapetalaceae | mph | GC | D | NA | Native |
| 167 | *Dichrostachys cinerea* **(L.) Wight & Arn.** | Fabaceae | mph | GC-SZ | D | LC | Exotic |
| 168 | *Digitaria argillacea* **(Hitchc.& Chase) Fern. f. argillacea** | Poaceae | Th | SZ | M | NA | Native |
| 169 | *Digitaria exilis* **(Kippist) Stapf** | Poaceae | He | SZ | M | NA | Native |
| 170 | *Digitaria horizontalis* **WillD** | Poaceae | He | GC-SZ | M | NA | Native |
| 171 | *Dioscorea abyssinica* **Hochst.** | Dioscoreaceae | Ge | GC-SZ | M | LC | Native |
| 172 | *Dioscorea bulbifera* **L.** | Dioscoreaceae | Ge | GC-SZ | M | NA | Native |
| 173 | *Dioscorea dumetorum* **(Kunth) Pax** | Dioscoreaceae | Ge | GC-SZ | M | NA | Native |
| 174 | *Dioscorea togoensis* **R. Knuth** | Dioscoreaceae | Ge | GC-SZ | M | NA | Native |
| 175 | *Diospyros mespiliformis* **Hochst.** | Ebenaceae | mPh | SZ | D | LC | Native |
| 176 | *Diospyros monbuttensis* **Gürke** | Ebenaceae | mph | GC | D | LC | Native |
| 177 | *Dissotis rotundifolia* **(Sm.) Triana** | Melastomataceae | Ch | GC | D | LC | Native |
| 178 | *Drypetes floribunda* **(Müll. Arg.) Hutch.** | Putranjivaceae | mph | GC | D | NA | Native |
| 179 | *Echinochloa colona* **(L.) Link** | Poaceae | Th | GC-SZ | M | LC | Exotic |
| 180 | *Ehretia cymosa* **Thonn.** | Ehretiaceae | mph | GC | D | LC | Native |
| 181 | *Elaeis guineensis* **Jacq.** | Arecaceae | mPh | GC | M | LC | Native |
| 182 | *Elaeophorbia drupifera* **(Thonn.) Stapf.** | Euphorbiaceae | mph | GC-SZ | D | NA | Native |
| 183 | *Elephantopus mollis* **Kunth** | Asteraceae | Th | GC | D | NA | Native |
| 184 | *Eleutheranthera ruderalis* **(Sw.) Sch.Bip.** | Asteraceae | Th | GC | D | NA | Native |
| 185 | *Emilia coccinea* **(Sims) G.Don** | Asteraceae | Th | GC | D | NA | Native |
| 186 | *Emilia sonchifolia* **(L.) DC.** | Asteraceae | Th | GC | D | NA | Native |
| 187 | *Entada abyssinica* **Steud.** | Fabaceae | mph | AT | D | LC | Native |
| 188 | *Eragrostis aspera* **(Jacq.) Nees** | Poaceae | Th | GC-SZ | M | NA | Native |
| 189 | *Eragrostis tremula* **Steud.** | Poaceae | Th | GC-SZ | M | NA | Native |
| 190 | *Eriosema psoraleoides* **(Lam.) G.Don** | Fabaceae | nph | GC-SZ | D | NA | Native |
| 191 | *Erythrina senegalensis* **DC.** | Fabaceae | mph | GC-SZ | D | LC | Native |
| 192 | *Erythrophleum suaveolens* **(Guill. & Perr.) Brenan** | Fabaceae | mPh | GC | D | NA | Native |
| 193 | *Eucalyptus camaldulensis* **Dehnh.** | Myrtaceae | mPh | I | D | NT | Exotic |
| 194 | *Euphorbia convolvuloides* **Hochst.** | Euphorbiaceae | Ch | AT | D | NA | Native |
| 195 | *Euphorbia heterophylla* **L.** | Euphorbiaceae | Th | GC-SZ | D | LC | Exotic |
| 196 | *Euphorbia hirta* **L.** | Euphorbiaceae | Th | GC-SZ | D | NA | Native |
| 197 | *Euphorbia hyssopifolia* **L.** | Euphorbiaceae | Th | GC-SZ | D | NA | Native |
| 198 | *Fadogia agrestis* **Schweif.ex Hiern** | Rubiaceae | Ch | SZ | D | NA | Native |
| 199 | *Feretia apodanthera* **Del.** | Rubiaceae | nph | SZ | D | NA | Native |
| 200 | *Ficus asperifolia* **Miq.** | Moraceae | nph | GC-SZ | D | LC | Native |
| 201 | *Ficus aurea* **Nutt.** | Moraceae | mph | GC-SZ | D | LC | Native |
| 202 | *Ficus auriculata* **Lour.** | Moraceae | mph | GC-SZ | D | LC | Native |
| 203 | *Ficus dicranostyla* **Mildbr.** | Moraceae | mph | GC-SZ | D | LC | Native |
| 204 | *Ficus elastica* **Roxb.** | Moraceae | mph | I | D | LC | Exotic |
| 205 | *Ficus exasperata* **Vahl** | Moraceae | mph | AT | D | LC | Native |
| 206 | *Ficus glumosa* **Delile** | Moraceae | mPh | SZ | D | LC | Native |
| 207 | *Ficus ingens* **(Miq.)** | Moraceae | mph | SZ | D | LC | Native |
| 208 | *Ficus lyrata* **Warb.** | Moraceae | mph | I | D | LC | Exotic |
| 209 | *Ficus mucuso* **Ficalho** | Moraceae | mPh | GC-SZ | D | LC | Native |
| 210 | *Ficus platyphylla* **Delile** | Moraceae | mph | GC-SZ | D | LC | Native |
| 211 | *Ficus polita* **Vahl** | Moraceae | mph | GC | D | LC | Native |
| 212 | *Ficus sur* **Forssk.** | Moraceae | mph | SG | D | LC | Native |
| 213 | *Ficus sycomorus* **L.** | Moraceae | mph | SZ | D | LC | Native |
| 214 | *Ficus vallis-choudae* **Delile** | Moraceae | mph | SZ | D | NA | Native |
| 215 | *Fimbristylis pilosa* **Vahl** | Cyperaceae | He | GC-SZ | M | NA | Native |
| 216 | *Flacourtia flavescens* **Willd.** | Salicaceae | mph | PRA | D | NA | Native |
| 217 | *Flueggea virosa* **(Roxb. ex Willd.) Royle** | Phyllantaceae | nph | Pan | D | LC | Exotic |
| 218 | *Funtumia africana* **(Benth.) Stapf** | Apocynaceae | mPh | GC | D | LC | Native |
| 219 | *Furcraea foetida* **(L.) Haw.** | Asparagaceae | mph | I | M | NA | Exotic |
| 220 | *Garcinia afzelii* **Engl.** | Clusiaceae | mph | GC-SZ | D | VU | Native |
| 221 | *Gardenia aqualla* **Stapf. & Hutch. aqualla** | Rubiaceae | nph | SZ | D | NA | Native |
| 222 | *Gardenia sokotensis* **Hutch.** | Rubiaceae | nph | SZ | D | LC | Native |
| 223 | *Gardenia ternifolia* **Schum. & Thonn.** | Rubiaceae | nph | Pal | D | LC | Native |
| 224 | *Gloriosa simplex* **L.** | Colchicaceae | Ge | SZ | D | NA | Native |
| 225 | *Glycine max* **(L.) Merr.** | Fabaceae | mph | I | D | NA | Exotic |
| 226 | *Gmelina arborea* **Roxb. ex Sm.** | Lamiaceae | mph | I | D | LC | Exotic |
| 227 | *Gomphrena celosioides* **C.Mart***.* | Amaranthaceae | Ch | GC-SZ | D | NA | Native |
| 228 | *Gomphrena globosa* **Linn.** | Amaranthaceae | Th | I | D | NA | Exotic |
| 229 | *Grewia mollis* **Juss.** | Malvaceae | nph | SZ | D | LC | Native |
| 230 | *Grewia venusta* **Fresen.** | Malvaceae | mph | SZ | D | NA | Native |
| 231 | *Griffonia simplicifolia* **(Vahl ex DC.) Baill.** | Fabaceae | Lmph | GC | D | NA | Native |
| 232 | *Gymnosporia senegalensis* **(Lam.)** | Celastraceae | nph | SZ | D | LC | Native |
| 233 | *Haematostaphis barteri* **Hook. F.** | Anacardiaceae | mph | SZ | D | NA | Native |
| 234 | *Hannoa undulata* **(Guill. & Perr.) Planch.** | Samaroubaceae | mPh | GC | D | NA | Native |
| 235 | *Harungana madagascariensis* **Lam. ex Poir.** | Hypericaceae | mph | GC | D | LC | Native |
| 236 | *Heteropogon contortus* **(L.) Roem. & Schult.** | Poaceae | He | GC-SZ | M | NA | Native |
| 237 | *Hexalobus monopetalus* **(A.Rich.) Engl. & Diels** | Annonaceae | mph | SZ | D | LC | Native |
| 238 | *Hibiscus asper* **Hook.f.** | Malvaceae | nph | GC-SZ | D | NA | Native |
| 239 | *Hibiscus cannabinus* **L.** | Malvaceae | Th | I | D | NA | Exotic |
| 240 | *Hibiscus esculentus* **L.** | Malvaceae | nph | GC-SZ | D | NA | Native |
| 241 | *Hibiscus rosa-sinensis* **L.** | Malvaceae | nph | I | D | NA | Exotic |
| 242 | *Hibiscus sabdariffa* **L.** | Malvaceae | nph | I | D | NA | Exotic |
| 243 | *Hibiscus scaber* **Lam.** | Malvaceae | nph | GC-SZ | D | NA | Native |
| 244 | *Hibiscus squamosus* **Hochr.** | Malvaceae | nph | GC-SZ | D | NA | Native |
| 245 | *Hibiscus surattensis* **L.** | Malvaceae | Lnph | GC-SZ | D | NA | Native |
| 246 | *Hibiscus syriacus* **L.** | Malvaceae | nph | GC-SZ | D | NA | Native |
| 247 | *Hippocratea indica* **Willd.** | Celastraceae | Lmph | GC | D | NA | Native |
| 248 | *Holarrhena floribunda* **(G. Don) Dur. & Schinz** | Apocynaceae | mPh | GC-SZ | D | LC | Native |
| 249 | *Hoslundia opposita* **Vahl** | Lamiaceae | nph | GC-SZ | D | NA | Native |
| 250 | *Hygrophila auriculata* **(Schumach.) Heine** | Acanthaceae | nph | GC-SZ | D | NA | Native |
| 251 | *Hymenocardia acida* **Tul.** | Phyllantaceae | mph | SZ | D | LC | Native |
| 252 | *Hyparrhenia involucrata* **Stapf** | Poaceae | Th | GC-SZ | M | NA | Native |
| 253 | *Hyparrhenia rufa* **(Nees) Stapf** | Poaceae | He | GC-SZ | M | NA | Exotic |
| 254 | *Hyptis spicigera* **Lam.** | Lamiaceae | nph | GC-SZ | D | NA | Native |
| 255 | *Hyptis suaveolens* **(L.) Poit.** | Lamiaceae | nph | GC-SZ | D | NA | Exotic |
| 256 | *Icacina senegalensis* **A. Juss.** | Icacinaceae | Lnph | GC | D | NA | Native |
| 257 | *Imperata cylindrica* **(L.) Raeusch.** | Poaceae | Ge | GC-SZ | M | LC | Exotic |
| 258 | *Indigofera dendroides* **Jacq.** | Fabaceae | nph | GC-SZ | D | NA | Native |
| 259 | *Indigofera hirsuta* **L.** | Fabaceae | nph | GC-SZ | D | NA | Native |
| 260 | *Indigofera lepreurii* **Bak.f.** | Fabaceae | nph | SZ | D | NA | Native |
| 261 | *Ipomoea aquatica* **Forssk.** | Convolvulaceae | Hyd | GC-SZ | D | LC | Native |
| 262 | *Ipomoea cairica* **(L.) Sweet** | Convolvulaceae | Lnph | GC-SZ | D | LC | Native |
| 263 | *Ipomoea eriocarpa* **R. Br.** | Convolvulaceae | Th | SZ | D | NA | Native |
| 264 | *Ipomoea mauritiana* **Jacq.** | Convolvulaceae | Lmph | GC-SZ | D | NA | Native |
| 265 | *Ipomoea obscura* **(L.) Ker-Gawl.** | Convolvulaceae | Th | GC | D | NA | Native |
| 266 | *Ipomoea triloba* **L.** | Convolvulaceae | Th | GC | D | NA | Native |
| 267 | *Isoberlinia doka* **Craib & Stapf** | Fabaceae | mph | SZ | D | LC | Native |
| 268 | *Isoberlinia tomentosa* **(Harms) Craib & Stapf** | Fabaceae | mph | SZ | D | LC | Native |
| 269 | *Justicia flava* **(Forssk.) Vahl** | Acanthaceae | nph | GC | D | VU | Native |
| 270 | *Kalanchoe pinnata* **(Lam.)** | Crassulaceae | nph | AT | D | NA | Native |
| 271 | *Khaya grandifoliola* **C. DC.** | Meliaceae | MPh | GC | D | VU | Native |
| 272 | *Khaya senegalensis* **(Desr.) A. Juss.** | Meliaceae | mPh | SZ | D | VU | Native |
| 273 | *Kigelia africana* **(Lam.) Benth.** | Bignoniaceae | mph | AT | D | LC | Native |
| 274 | *Kyllinga bulbosa* **P.Beauv.** | Cyperaceae | Ge | AT | M | LC | Native |
| 275 | *Lannea acida* **A. Rich.** | Anacardiaceae | mPh | PRA | D | LC | Native |
| 276 | *Lannea barteri* **(Oliv.) Engl.** | Anacardiaceae | mPh | PRA | D | LC | Native |
| 277 | *Lannea microcarpa* **Engl. & K. Krause** | Anacardiaceae | mPh | SZ | D | LC | Native |
| 278 | *Lantana rhodesiensis* **Moldenke** | Verbenaceae | Ch | Pan | D | NA | Native |
| 279 | *Launaea taraxacifolia* **(Willd.) Amin ex C.Jeffrey** | Asteraceae | Th | GC-SZ | D | NA | Native |
| 280 | *Lecaniodiscus cupanioides* **Planch.** | Sapindaceae | mph | GC | D | LC | Native |
| 281 | *Leersia hexandra* **Sw.** | Poaceae | He | GC-SZ | M | LC | Native |
| 282 | *Leucaena leucocephala* **(Lam.) de Wit** | Fabaceae | mph | GC-SZ | D | CD | Exotic |
| 283 | *Leucas martinicensis* **(Jacq.) R. Br.** | Lamiaceae | Th | GC-SZ | D | NA | Native |
| 284 | *Lonchocarpus sericeus* **(Poir.) Kunth ex DC.** | Fabaceae | mPh | PRA | D | LC | Native |
| 285 | *Lophira lanceolata* **Van Tiegh.** | Ochnaceae | mph | SZ | D | LC | Native |
| 286 | *Loudetia phragmitoides* **(Peter) CE Hubbard** | Poaceae | He | GC-SZ | M | LC | Native |
| 287 | *Loudetia simplex* **(Nees) C.E.Hubb***.* | Poaceae | He | SZ | M | NA | Native |
| 288 | *Ludwigia erecta* **(L.) H.Hara** | Onagraceae | Th | GC-SZ | D | NA | Native |
| 289 | *Ludwigia hyssopifolia* **(G. Don) Exell** | Onagraceae | Th | Pal | D | LC | Native |
| 290 | *Macaranga barteri* **Müll.Arg.** | Euphorbiaceae | mph | GC | D | LC | Native |
| 291 | *Macrosphyra longistyla* **L.** | Rubiaceae | Lmph | GC | D | NA | Native |
| 292 | *Mallotus oppositifolius* **(Geiseler) Müll. Arg** | Euphorbiaceae | nph | GC-SZ | D | LC | Native |
| 293 | *Mangifera indica* **L.** | Anacardiaceae | mPh | Pan | D | DD | Exotic |
| 294 | *Manihot esculenta* **Crantz** | Euphorbiaceae | mph | I | D | DD | Exotic |
| 295 | *Manilkara multinervis* **(Bak.) Dubard** | Sapotaceae | mph | GC-SZ | D | NA | Native |
| 296 | *Maranthes polyandra* **(Benth.) Prance** | Chrysobalanaceae | mph | SZ | D | LC | Native |
| 297 | *Marantochloa purpurea* **(Ridl.) Milne-Redh.** | Maranthaceae | nph | GC | D | LC | Native |
| 298 | *Margaritaria discoidea* **(Baill.) G.L.Webster** | Euphorbiaceae | mPh | GC-SZ | D | NA | Native |
| 299 | *Mariscus cylindristachyus* **Steud.** | Cyperaceae | He | GC | M | NA | Native |
| 300 | *Maytenus senegalensis* **(Lam.) Exell** | Celastraceae | mph | SZ | D | NA | Native |
| 301 | *Melanthera scandens* **(Schum. & Thonn.)** | Asteraceae | Lnph | GC | D | NA | Native |
| 302 | *Melinis repens* **(Willd.) Zizka** | Poaceae | Th | SZ | M | NA | Native |
| 303 | *Mezoneuron benthamianum* **Baill.** | Fabaceae | Lmph | GC | D | NA | Native |
| 304 | *Mikania chenopodiifolia* **Willd.** | Asteraceae | Lnph | GC | D | NA | Native |
| 305 | *Milicia excelsa (Welw.)* **C.C. Berg** | Moraceae | mPh | GC | D | NT | Native |
| 306 | *Millettia thonningii* **(Schumach. & Thonn.) Baker** | Fabaceae | mph | GC | D | LC | Native |
| 307 | *Millettia zechiana* **Harms** | Fabaceae | mph | GC | D | LC | Native |
| 308 | *Mimosa pudica* **L.** | Fabaceae | Th | I | D | LC | Exotic |
| 309 | *Mimusops andongensis* **Hiern** | Sapotaceae | mph | GC-SZ | D | LC | Native |
| 310 | *Mimusops kummel* **Bruce ex A.DC.** | Sapotaceae | mPh | SZ | D | LC | Native |
| 311 | *Mitracarpus scaber* **Zucc. ex Schult. & Schult. f.** | Rubiaceae | Th | GC-SZ | D | NA | Native |
| 312 | *Mitragyna inermis* **(Willd.) O Ktze.** | Rubiaceae | mph | SZ | D | LC | Native |
| 313 | *Mnesithea granularis* **L.** | Poaceae | Th | GC-SZ | M | NA | Native |
| 314 | *Mollugo nudicaulis* **Lam.** | Molluginaceae | Th | GC-SZ | D | NA | Native |
| 315 | *Momordica charantia* **L.** | Cucurbitaceae | Lmph | GC-SZ | D | NA | Native |
| 316 | *Monechma depauperatum* **(T. Anders.) C.B.Cl.** | Acanthaceae | nph | SZ | D | NA | Native |
| 317 | *Monodora myristica* **(Gaertn.) Dunal** | Annonaceae | mPh | GC | D | LC | Native |
| 318 | *Monotes kerstingii* **Gilg** | Dipterocarpaceae | mph | SZ | M | LC | Native |
| 319 | *Morelia senegalensis* **A Rich.** | Rubiaceae | mph | GC | D | LC | Native |
| 320 | *Morinda lucida* **Benth.** | Rubiaceae | mph | GC-SZ | D | LC | Native |
| 321 | *Mucuna poggei* **Taub.** | Fabaceae | Lmph | GC-SZ | D | NA | Native |
| 322 | *Mucuna pruriens* **(L.) DC.** | Fabaceae | Lmph | GC-SZ | D | LC | Native |
| 323 | *Musa sapientum* **Linn.** | Musaceae | He | I | M | LC | Exotic |
| 324 | *Musanga cecropioides* **R. Br.** | Urticaceae | mph | GC | D | LC | Native |
| 325 | *Nauclea latifolia* **Sm.** | Rubiaceae | mph | GC-SZ | D | LC | Native |
| 326 | *Nephrolepis undulata* **(Afzel. ex Sw.) J. Sm.** | Nephrolepidaceae | He | GC-SZ | P | LC | Native |
| 327 | *Newbouldia laevis* **Seem.** | Bignoniaceae | mph | GC | D | LC | Native |
| 328 | *Nymphaea lotus* **L.** | Nympheaceae | Hyd | GC-SZ | D | LC | Native |
| 329 | *Ochna afzelii* **R.Br.** | Ochnaceae | nph | GC-SZ | D | LC | Native |
| 330 | *Ochna membranacea* **Oliv.** | Ochnaceae | mph | AT | D | LC | Native |
| 331 | *Ochna schweinfurthiana* **F. Hoffm.** | Ochnaceae | nph | SZ | D | LC | Native |
| 332 | *Ocimum americanum* **L.** | Lamiaceae | nph | Pan | D | NA | Native |
| 333 | *Oldenlandia corymbosa* **L.** | Rubiaceae | Ch | Pan | D | LC | Native |
| 334 | *Opilia amentacea* **Roxb.** | Opiliaceae | Lmph | SZ | D | NA | Native |
| 335 | *Oryza sativa* **L.** | Poaceae | Th | I | M | LC | Exotic |
| 336 | *Oxytenanthera abyssinica* **(A. Rich.) Munro** | Poaceae | mph | SZ | M | NA | Native |
| 337 | *Pandanus candelabrum* **P. Beauv.** | Pandanaceae | mph | GC-SZ | M | LC | Native |
| 338 | *Pandiaka angustifolia* **(Vahl) Hepper** | Amaranthaceae | Th | GC-SZ | D | NA | Native |
| 339 | *Pandiaka involucrata* **(Moq.) Hook. f.** | Amaranthaceae | Th | GC-SZ | D | NA | Native |
| 340 | *Panicum maximum* **Jacq.** | Poaceae | He | GC | M | NA | Native |
| 341 | *Parinari congensis* **F. Didr.** | Chrysobalanaceae | MPh | GC | D | LC | Native |
| 342 | *Parinari curatellifolia* **Planch.** | Chrysobalanaceae | mph | SZ | D | LC | Native |
| 343 | *Parkia biglobosa* **(Jacq.) R.Br. ex G.Don** | Fabaceae | MPh | Pal | D | LC | Native |
| 344 | *Parkia filicoidea* **Welw. ex Oliv.** | Fabaceae | MPh | GC | D | LC | Native |
| 345 | *Parquetina nigrescens* **(Afzel.) Bullock.** | Apocynaceae | Lmph | GC | D | NA | Native |
| 346 | *Paspalum scrobiculatum* **L.** | Poaceae | He | SZ | M | LC | Native |
| 347 | *Paspalum vaginatum* **Sw.** | Poaceae | He | SZ | M | LC | Native |
| 348 | *Passiflora foetida* **L.** | Passifloraceae | Lmph | GC-SZ | D | NA | Exotic |
| 349 | *Paullinia pinnata* **L.** | Sapindaceae | Lmph | GC-SZ | D | NA | Native |
| 350 | *Pavetta corymbosa* **(DC.) F.N.Williams** | Rubiaceae | mph | GC-SZ | D | LC | Native |
| 351 | *Pavetta crassipes* **K.Schum.** | Rubiaceae | nph | SZ | D | LC | Native |
| 352 | *Pennisetum pedicellatum* **Trin.** | Poaceae | Th | GC-SZ | M | LC | Native |
| 353 | *Pennisetum polystachion* **(L.) Schult.** | Poaceae | Th | GC-SZ | M | LC | Exotic |
| 354 | *Pennisetum purpureum* **Schumach.** | Poaceae | Th | SZ | M | LC | Native |
| 355 | *Pennisetum violaceum* **(Lam.) Rich.** | Poaceae | Th | GC-SZ | M | LC | Native |
| 356 | *Pentadesma butyracea* **Sab.** | Clusiaceae | mPh | GC-SZ | D | LC | Native |
| 357 | *Pentanema indicum* **(L.)** | Asteraceae | Th | Pal | D | NA | Native |
| 358 | *Pergularia daemia* **(Forssk.) Chiov.** | Apocynaceae | Lmph | GC | D | LC | Native |
| 359 | *Pericopsis laxiflora* **(Benth.) Meeuwen** | Fabaceae | mPh | SG | D | LC | Native |
| 360 | *Perotis indica* **(L.) Kuntze** | Poaceae | Th | GC | M | NA | Native |
| 361 | *Persea americana* **Mill.** | Lauraceae | mph | I | D | LC | Exotic |
| 362 | *Phaulopsis barteri* **T. Anders.** | Acanthaceae | nph | GC-SZ | D | NA | Native |
| 363 | *Phaulopsis imbricata* **(Forssk.) Sweet** | Acanthaceae | nph | GC-SZ | D | LC | Native |
| 364 | *Phoenix reclinata* **Jacq.** | Arecaceae | mph | GC-SZ | M | LC | Native |
| 365 | *Phyllanthus amarus* **Schum. & Thonn.** | Phyllantaceae | Th | GC | D | NA | Native |
| 366 | *Phyllanthus muellerianus* **(O Ktze) Exell** | Phyllantaceae | nph | GC-SZ | D | NA | Native |
| 367 | *Phyllanthus niruri* **L.** | Phyllantaceae | nph | GC | D | NA | Native |
| 368 | *Phyllanthus pentandrus* **Schumach. & Thonn.** | Phyllantaceae | nph | GC-SZ | D | NA | Native |
| 369 | *Physalis angulata* **L.** | Solanaceae | Th | GC-SZ | D | LC | Exotic |
| 370 | *Physalis micrantha* **Link.** | Solanaceae | Th | GC-SZ | D | NA | Native |
| 371 | *Piliostigma thonningii* **(Schumach.) Milne-Redh.** | Fabaceae | mph | GC-SZ | D | LC | Native |
| 372 | *Piper guineense* **Schumach. & Thonn.** | Piperaceae | Lmph | GC | D | LC | Native |
| 373 | *Piptadeniastrum africanum* **(Hook. f.) Brenan** | Fabaceae | MPh | GC | D | LC | Native |
| 374 | *Polygala myrtifolia* **L.** | Polygalaceae | Th | SZ | D | NA | Native |
| 375 | *Polygonum senegalense* **Meisn.** | Polygalaceae | nph | GC-SZ | D | LC | Native |
| 376 | *Polysphaeria arbuscula* **K.Schum.** | Rubiaceae | mph | SZ | D | LC | Native |
| 377 | *Porophyllum ruderale* **(Jacq.) Cass.** | Asteraceae | He | Pan | D | NA | Exotic |
| 378 | *Pouteria alnifolia* **(Baker) Roberty** | Sapotaceae | mPh | GC | D | LC | Native |
| 379 | *Premna quadrifolia* **Schumach. & Thonn.** | Lamiaceae | Lnph | GC | D | LC | Native |
| 380 | *Prosopis africana* **(Guill. & Perr.) Taub.** | Fabaceae | mPh | SZ | D | LC | Native |
| 381 | *Pseudocedrela kotschyi* **(Schweinf.) Harms** | Meliaceae | mph | SZ | D | LC | Native |
| 382 | *Pseudospondias microcarpa* **Engl.** | Anacardiaceae | mPh | GC | D | LC | Native |
| 383 | *Psidium guajava* **L.** | Myrtaceae | mph | I | D | LC | Exotic |
| 284 | *Psorospermum ferrugineum* **Hook. f.** | Hypericaceae | mph | SZ | D | NA | Native |
| 385 | *Psychotria peduncularis* **(Salisb.) Steyerm** | Rubiaceae | nph | GC | D | LC | Native |
| 386 | *Pteleopsis suberosa* **Engl. & Diels** | Combretaceae | mph | PRA | D | LC | Native |
| 387 | *Pterocarpus erinaceus* **Poir.** | Fabaceae | mPh | SZ | D | EN | Native |
| 388 | *Pterocarpus santalinoides* **L'Hér.ex DC.** | Fabaceae | mPh | PRA | D | LC | Native |
| 389 | *Pupalia lappacea* **(L.) Juss.** | Amaranthaceae | nph | Pal | D | LC | Native |
| 390 | *Pycnanthus angolensis* **(Welw.) Warb.** | Myristicaceae | MPh | GC | D | LC | Native |
| 391 | *Raphia sudanica* **A.Chev.** | Arecaceae | mph | SZ | M | NT | Native |
| 392 | *Rauvolfia vomitoria* **Afzel.** | Apocynaceae | mph | GC-SZ | D | LC | Native |
| 393 | *Rhizophora racemosa* **G.Mey.** | Rhizophoraceae | mph | GC | D | LC | Native |
| 394 | *Rhynchelytrum repens* **(Willd.) Hubb.** | Poaceae | Th | SZ | M | NA | Native |
| 395 | *Ricinodendron heudelotii* **(Baill.) Pierre ex Heckel** | Euphorbiaceae | mPh | GC | D | LC | Native |
| 396 | *Ritchiea reflexa* **(Thonn.) Gilg & Gilg-Ben.** | Capparaceae | Lnph | GC-SZ | D | NA | Native |
| 397 | *Rothmannia longiflora* **Salisb.** | Rubiaceae | mph | GC | D | LC | Native |
| 298 | *Rottboellia exaltata* **L. f.** | Poaceae | Th | SZ | M | NA | Native |
| 399 | *Rourea coccinea* **(Thonn. ex Schumach.) Benth.** | Connaraceae | mph | GC | D | NA | Native |
| 400 | *Rytigynia umbellulata* **(Hiern) Robyns** | Rubiaceae | mph | GC | D | NA | Native |
| 401 | *Saba comorensis* **(Bojer) Pichon** | Apocynaceae | Lmph | GC | D | NA | Native |
| 402 | *Sansevieria cylindrica* **Boj.** | Asparagaceae | Ge | GC-SZ | M | NA | Native |
| 403 | *Sarcocephalus latifolius* **(Sm.) E.A.Bruce** | Rubiaceae | mph | AT | D | NA | Native |
| 404 | *Schrankia leptocarpa* **DC.** | Fabaceae | nph | GC-SZ | D | NA | Native |
| 405 | *Schwenckia americana* **D.Royen ex L.** | Solanaceae | Th | GC-SZ | D | NA | Native |
| 406 | *Scleria depressa* **(C.B.Clarke) Nelmes** | Cyperaceae | Ge | GC | M | LC | Native |
| 407 | *Sclerocarya birrea* **(A.Rich.) Hochst.** | Anacardiaceae | mPh | SZ | D | LC | Native |
| 408 | *Scoparia dulcis* **L.** | Plantaginaceae | Ch | GC-SZ | D | NA | Native |
| 409 | *Sebastiania chamaelea* **(L.) Müll.Arg.** | Euphorbiaceae | Lnph | GC | D | NA | Native |
| 410 | *Secamone afzelii* **(Schult.) K. Schum.** | Apocynaceae | Lmph | GC | D | NA | Native |
| 411 | *Securidaca longipedunculata* **Fresen.** | Polygalaceae | mph | SZ | D | NA | Native |
| 412 | *Senna hirsuta* **(L.) H.S.Irwin & Barneby** | Fabaceae | nph | GC-SZ | D | NA | Native |
| 413 | *Senna obtusifolia* **(L.) Irwin & Barneby** | Fabaceae | nph | Pan | D | NA | Native |
| 414 | *Senna siamea* **(Lam.) H.S. Irwin et Barneby.** | Fabaceae | mph | I | D | LC | Exotic |
| 415 | *Senna tora* **(L.) Roxb.** | Fabaceae | nph | Pan | D | NA | Native |
| 416 | *Sesbania rostrata* **Bremek. & Oberm.** | Fabaceae | Th | GC | D | LC | Native |
| 417 | *Setaria barbata* **(Lam.) Kunth** | Poaceae | He | Pan | M | NA | Exotic |
| 418 | *Setaria megaphylla* **(Steud.) T.Durand & Schinz** | Poaceae | He | GC | M | NA | Native |
| 419 | *Sida acuta* **Burm.f.** | Malvaceae | nph | GC-SZ | D | NA | Exotic |
| 420 | *Sida cordifolia* **L.** | Malvaceae | nph | GC | D | NA | Native |
| 421 | *Sida linifolia* **Juss. ex Cav.** | Malvaceae | nph | GC | D | NA | Native |
| 422 | *Sida rhombifolia* **L.** | Malvaceae | nph | GC | D | NA | Native |
| 423 | *Sida urens* **L.** | Malvaceae | nph | GC | D | NA | Native |
| 424 | *Smilax kraussiana* **Meisn.** | Smilacaceae | Lmph | GC-SZ | D | NA | Native |
| 425 | *Solenostemon monostachyus* **(P.Beauv.) Briq.** | Lamiaceae | nph | SZ | D | NA | Native |
| 426 | *Sonchus oleraceus* **L.** | Asteraceae | Th | Cosm | D | LC | Native |
| 427 | *Sorghum arundinaceum* **(Desv.) Stapf.** | Poaceae | He | GC-SZ | M | LC | Exotic |
| 428 | *Sorghum bicolor* **(L.) Moench** | Poaceae | He | SZ | M | LC | Native |
| 429 | *Sorindeia warneckei* **Engl.** | Anacardiaceae | nph | GC | D | NA | Native |
| 430 | *Spathodea campanulata* **P.Beauv.** | Bignoniaceae | mPh | GC | D | LC | Native |
| 431 | *Spermacoce radiata* **(DC.) Hiern** | Rubiaceae | Th | GC-SZ | D | LC | Native |
| 432 | *Spermacoce ruelliae* **DC.** | Rubiaceae | Th | GC-SZ | D | NA | Native |
| 433 | *Spigelia anthelmia* **L.** | Loganiaceae | Th | GC-SZ | D | NA | Exotic |
| 434 | *Spilanthes costata* **Benth.** | Asteraceae | Ch | GC-SZ | D | NA | Native |
| 435 | *Spilanthes oleracea* **L.** | Asteraceae | Th | GC-SZ | D | NA | Native |
| 436 | *Spondias mombin* **L.** | Anacardiaceae | mPh | I | D | LC | Exotic |
| 437 | *Sporobolus pyramidalis* **P.Beauv.** | Poaceae | He | SZ | M | NA | Native |
| 438 | *Sporobolus robusta* **Kunth** | Poaceae | He | SZ | M | NA | Native |
| 439 | *Steganotaenia araliacea* **Hochst.** | Apiaceae | mph | GC-SZ | D | LC | Native |
| 440 | *Sterculia setigera* **Delile** | Malvaceae | mph | SZ | D | LC | Native |
| 441 | *Sterculia tragacantha* **Lindl.** | Malvaceae | mph | AT | D | LC | Native |
| 442 | *Stereospermum kunthianum* **Cham.** | Bignoniaceae | mph | SG | D | LC | Native |
| 443 | *Strychnos afzelii* **Gilg** | Loganiaceae | LMPh | GC | D | NA | Native |
| 444 | *Strychnos innocua* **Del.** | Loganiaceae | mph | SZ | D | LC | Native |
| 445 | *Strychnos spinosa* **Lam***.* | Loganiaceae | mPh | SZ | D | LC | Native |
| 446 | *Stylochaeton hypogaeus* **Lepr.** | Araceae | Ge | SZ | M | NA | Native |
| 447 | *Stylochaeton lancifolius* **Kotschy & Peyr.** | Araceae | Ge | SZ | M | NA | Native |
| 448 | *Synedrella nodiflora* **(L.) Gaertn.** | Asteraceae | Th | Pan | D | NA | Native |
| 449 | *Syzygium guineense* **(Willd.) DC.** | Myrtaceae | mph | SZ | D | LC | Native |
| 450 | *Tabernaemontana pachysiphon* **Stapf** | Apocynaceae | mPh | GCW | D | LC | Native |
| 451 | *Tacca leontopetaloides* **(L.) Kuntze** | Taccaceae | Ge | GC-SZ | D | LC | Native |
| 452 | *Talinum triangulare* **(Jacq.) Willd.** | Talinaceae | nph | GC | D | NA | Native |
| 453 | *Tamarindus indica* **L.** | Fabaceae | mPh | Pan | D | LC | Native |
| 454 | *Tectona grandis* **L. f.** | Verbenaceae | mPh | I | D | EN | Exotic |
| 455 | *Tephrosia bracteolata* **Guill. & Perr.** | Fabaceae | Th | SZ | D | NA | Native |
| 456 | *Tephrosia elegans* **Schumach.** | Fabaceae | nph | GC-SZ | D | NA | Native |
| 457 | *Tephrosia flexuosa* **G.Don** | Fabaceae | nph | GC-SZ | D | NA | Native |
| 458 | *Terminalia avicennioides* **Guill. & Perr.** | Combretaceae | mph | SZ | D | LC | Native |
| 459 | *Terminalia glaucescens* **Planch. ex Benth.** | Combretaceae | mph | SZ | D | LC | Native |
| 460 | *Terminalia laxifolia* **Engl.** | Combretaceae | mph | SZ | D | NA | Native |
| 461 | *Terminalia macroptera* **Guill. ex Perr.** | Combretaceae | mph | SZ | D | LC | Native |
| 462 | *Terminalia mollis* **Laws.** | Combretaceae | mph | SZ | D | LC | Native |
| 463 | *Terminalia superba* **Engl. & Diels** | Combretaceae | MPh | GC | D | NA | Native |
| 464 | *Theobroma cacao* **L.** | Malvaceae | mph | I | D | NA | Exotic |
| 465 | *Tiliacora funifera* **Oliv.** | Menispermaceae | Lmph | GC-SZ | D | NA | Native |
| 466 | *Tithonia diversifolia* **(Hemsl.) A. Gray.** | Asteraceae | nph | I | D | NA | Exotic |
| 467 | *Tragia benthamii* **Baker** | Euphorbiaceae | Lnph | GC | D | NA | Native |
| 468 | *Trema orientalis* **(L.) Blume** | Cannabaceae | mph | GC-SZ | D | LC | Native |
| 469 | *Trichilia emetica* **Vahl** | Meliaceae | mph | GC | D | NA | Native |
| 470 | *Triclisia subcordata* **Oliv.** | Menispermaceae | Lmph | GC-SZ | D | NA | Native |
| 471 | *Tridax procumbens* **L.** | Asteraceae | Th | Pan | D | NA | Exotic |
| 472 | *Trilepisium madagascariense* **DC.** | Moraceae | MPh | GC | D | NA | Native |
| 473 | *Triumfetta cordifolia* **A. Rich.** | Malvaceae | nph | GC | D | NA | Native |
| 474 | *Triumfetta rhomboidea* **Jacq.** | Malvaceae | nph | GC-SZ | D | NA | Native |
| 475 | *Uapaca guineensis* **Müll.Arg.** | Phyllantaceae | mPh | GC | D | LC | Native |
| 476 | *Uraria picta* **(Jacq.) Desv.** | Fabaceae | Th | Pal | D | LC | Native |
| 477 | *Urena lobata* **L.** | Malvaceae | nph | Pan | D | LC | Native |
| 478 | *Uvaria chamae* **P. Beauv.** | Annonaceae | Lmph | GC-SZ | D | LC | Native |
| 479 | *Vangueria madagascariensis* **J.F. Gmel.** | Rubiaceae | mph | GCW | D | LC | Native |
| 480 | *Vernonia ambigua* **Kotschy & Peyr** | Asteraceae | nph | SZ | D | NA | Native |
| 481 | *Vernonia amygdalina* **Delile (A–C).** | Asteraceae | nph | GC | D | NA | Native |
| 482 | *Vernonia cinerea* **(L.)** | Asteraceae | nph | GC-SZ | D | NA | Native |
| 483 | *Vernonia colorata* **(Willd.) Drake** | Asteraceae | nph | GC-SZ | D | LC | Native |
| 484 | *Vernonia pauciflora* **(Willd.) Less.** | Asteraceae | Th | SZ | D | NA | Native |
| 485 | *Vigna oblongifolia* **A.Rich.** | Fabaceae | Lnph | GC-SZ | D | LC | Native |
| 486 | *Vigna unguiculata* **(L.) Walp.** | Fabaceae | Lmph | GC-SZ | D | NA | Native |
| 487 | *Vitellaria paradoxa* **C.F.Gaertn. ssp. paradoxa** | Sapotaceae | mPh | SG | D | VU | Exotic |
| 488 | *Vitex doniana* **Sweet** | Lamiaceae | mPh | SZ | D | LC | Native |
| 489 | *Voacanga africana* **Stapf** | Apocynaceae | mph | GC | D | LC | Native |
| 490 | *Waltheria indica* **L.** | Malvaceae | Ch | Pan | D | LC | Native |
| 491 | *Wissadula amplissima* **(L.) R.E.Fr.** | Malvaceae | nph | GC-SZ | D | NA | Native |
| 492 | *Xanthosoma mafaffa* **Schott** | Araceae | He | I | D | NA | Exotic |
| 493 | *Xeroderris stuhlmannii***(Taub.) Mendonca & Sousa** | Fabaceae | mph | SZ | D | NA | Native |
| 494 | *Xylopia aethiopica* **(Dunal) A. Rich.** | Annonaceae | mPh | GC | D | LC | Native |
| 495 | *Zanha golungensis* **Hiern** | Sapindaceae | mph | GC-SZ | D | NA | Native |
| 496 | *Zanthoxylum zanthoxyloides* **(Lam.) Zepern. & Timler** | Rutaceae | mph | GC-SZ | D | LC | Native |
| 497 | *Zea mays* **L.** | Poaceae | Th | I | M | LC | Exotic |
| 498 | *Ziziphus mucronata* **Willd.** | Rhamnaceae | mph | SZ | D | LC | Native |

Appendix B: List of Threatened Species, Invasive and/or Alien Species, Agroforestry Species, and Soil-Fertility-Enhancing Species

| N° | Species | Family | Invasive and/or Alien Species | Agroforestry Species | Soil-Fertility-Enhancing | Ecological zone |
| --- | --- | --- | --- | --- | --- | --- |
| 1 | *Acacia ataxacantha* **DC.** | Fabaceae |  |  | Yes | IV |
| 2 | *Acacia auriculiformis* **A. Cunn. ex Benth**. | Fabaceae |  |  | Yes | III,V |
| 3 | *Acacia gourmaensis* **A Chev.** | Fabaceae |  | Yes | Yes | I |
| 4 | *Acacia macrostachya* **Reichenb.** | Fabaceae |  |  | Yes | I |
| 5 | *Acacia nilotica* **(L.) Willd.** | Fabaceae |  | Yes | Yes | V |
| 6 | *Acacia polyacantha* **Willd.** | Fabaceae |  | Yes | Yes | I, III, V |
| 7 | *Acacia seyal* **DC.** | Fabaceae |  | Yes | Yes | I |
| 8 | *Acacia sieberiana* **DC.** | Fabaceae |  | Yes | Yes | I, II, III, V |
| 9 | *Acanthospermum hispidum* **DC.** | Asteraceae | Yes |  |  | I, V |
| 10 | *Adansonia digitata* **L.** | Malvaceae |  | Yes |  | I, V |
| 11 | *Afzelia africana* **Sm.** | Fabaceae |  | Yes |  | I, II, III |
| 12 | *Ageratum conyzoides* **L.** | Asteraceae | Yes |  |  | I,II, III, IV, V |
| 13 | *Albizia adianthifolia* **(Schumach.) W.Wight** | Fabaceae |  | Yes | Yes | III,IV |
| 14 | *Albizia chevalieri* **Harms** | Fabaceae |  | Yes | Yes | I, III, V |
| 15 | *Albizia ferruginea* (Guill. & Perr.) Benth. | Fabaceae |  | Yes | Yes | I, III, IV |
| 16 | *Albizia lebbeck* **(L.) Benth.** | Fabaceae |  | Yes | Yes | II |
| 17 | *Albizia zygia* **(DC.) J.F. Macbr.** | Fabaceae |  | Yes | Yes | I, II, III, IV, V |
| 18 | *Andropogon africanus* **Franch.** | Poaceae | Yes |  |  | I, II, III |
| 19 | *Andropogon chinensis* **(Nees) Merr.** | Poaceae | Yes |  |  | I |
| 20 | *Andropogon contortus* **L.** | Poaceae | Yes |  |  | II |
| 21 | *Andropogon gayanus* **kunth var. gayanus** | Poaceae | Yes |  |  | I,II, III, V |
| 22 | *Andropogon tectorum* **Schum. & Thonn.** | Poaceae | Yes |  |  | I,II, III, V |
| 23 | *Azadirachta indica* **A. Juss.** | Meliaceae | Yes |  |  | I, III, IV, V |
| 24 | *Cajanus cajan* **(L.) Millsp.** | Fabaceae |  |  | Yes | III |
| 25 | *Calopogonium mucunoides* **pesv.** | Fabaceae | Yes |  | Yes | II, III, V |
| 26 | *Chromolaena odorata* **(L.) R.M.King & H.Rob.** | Asteraceae | Yes |  |  | I, II, III, IV, V |
| 27 | *Citrus limon* **(L.) Burm. f.** | Rutaceae |  |  | Yes | V |
| 28 | *Citrus sinensis* **(L.)** | Rutaceae |  |  | Yes | IV, V |
| 29 | *Coffea arabica* **L.** | Rubiaceae |  | Yes |  | I, II, IV |
| 30 | *Coffea canephora* **Pierre ex Froehner** | Rubiaceae |  | Yes |  | III |
| 31 | *Commelina aspera* **Benth.** | Commelinaceae | Yes |  |  | II |
| 32 | *Commelina benghalensis* **L**. | Commelinaceae | Yes |  |  | III, IV |
| 33 | *Commelina erecta* **L. subsp. Erecta** | Commelinaceae | Yes |  |  | I, II, III, IV, V |
| 34 | *Dactyloctenium aegyptium* **(L.) Willd.** | Poaceae | Yes |  |  | I, III, V |
| 35 | *Desmodium tortuosum* **(Sw.) DC.** | Fabaceae |  |  | Yes | III, V |
| 36 | *Dialium guineense* **Willd.** | Fabaceae |  | Yes |  | I, II, III, V |
| 37 | *Digitaria horizontalis* **Willd** | Poaceae | Yes |  |  | I, III,V |
| 38 | *Elaeis guineensis* **Jacq.** | Arecaceae |  | Yes |  | II, III, IV |
| 39 | *Euphorbia heterophylla* **L.** | Euphorbiaceae | Yes |  |  | III, V |
| 40 | *Euphorbia hirta* **L** | Euphorbiaceae | Yes |  |  | I, II, III |
| 41 | *Flueggea virosa* **(Roxb. ex Willd.) Royle** | Phyllantaceae | Yes |  |  | I, II, III, V |
| 42 | *Glycine max* **(L.) Merr.** | Fabaceae |  |  | Yes | III, IV |
| 43 | *Hyparrhenia involucrata* **Stapf** | Poaceae | Yes |  |  | I |
| 44 | *Hyparrhenia rufa* **(Nees) Stapf** | Poaceae | Yes |  |  | I, II |
| 45 | *Hyptis spicigera* **Lam.** | Lamiaceae | Yes |  |  | I, III |
| 46 | *Hyptis suaveolens* **(L.) Poit.** | Lamiaceae | Yes |  |  | I, III, IV, V |
| 47 | *Imperata cylindrica* **(L.) Raeusch.** | Poaceae | Yes |  |  | I, III, IV |
| 48 | *Leucaena leucocephala* **(Lam.) de Wit** | Fabaceae |  | Yes | Yes | I, II, III, IV, V |
| 49 | *Mangifera indica* **L.** | Anacardiaceae |  | Yes |  | I, II, III, V |
| 50 | *Melinis repens* **(Willd.) Zizka** | Poaceae | Yes |  |  | III, IV, V |
| 51 | *Musa sapientum* **Linn.** | Musaceae |  | Yes |  | IV |
| 52 | *Panicum maximum* **Jacq.** | Poaceae | Yes |  |  | I, III, IV, V |
| 53 | *Parkia biglobosa* **(Jacq.) R.Br. ex G.Don** | Fabaceae |  | Yes |  | I, II, III, IV, V |
| 54 | *Paspalum scrobiculatum* **L**. | Poaceae | Yes |  |  | I, II, III, V |
| 55 | *Paspalum vaginatum* **Sw.** | Poaceae | Yes |  |  | IV, V |
| 56 | *Passiflora foetida* **L.** | Passifloraceae | Yes |  |  | V |
| 57 | *Pennisetum pedicellatum* **Trin.** | Poaceae | Yes |  |  | I, II, III |
| 58 | *Pennisetum polystachion* **(L.) Schult.** | Poaceae | Yes |  |  | I, II, III, V |
| 59 | *Pennisetum purpureum* **Schumach.** | Poaceae | Yes |  |  | III |
| 60 | *Pennisetum violaceum* **(Lam.) Rich.** | Poaceae | Yes |  |  | I |
| 61 | *Persea americana* **Mill.** | Lauraceae |  | Yes |  | IV |
| 62 | *Piper guineense* **Schumach. & Thonn.** | Piperaceae |  | Yes |  | IV |
| 63 | *Psidium guajava* **L.** | Myrtaceae |  | Yes |  | I, II, V |
| 64 | *Pupalia lappacea* **(L.) Juss.** | Amaranthaceae | Yes |  |  | V |
| 65 | *Rothmannia longiflora* **Salisb.** | Rubiaceae | Yes |  |  | IV |
| 66 | *Rottboellia exaltata* **L. f.** | Poaceae | Yes |  |  | I, II, III, IV, V |
| 67 | *Securidaca longipedunculata* **Fresen.** | Polygalaceae |  | Yes |  | I, II, III |
| 68 | *Senna obtusifolia* **(L.) Irwin & Barneby** | Fabaceae | Yes |  |  | V |
| 69 | *Sida acuta* **Burm.f.** | Malvaceae | Yes |  |  | I, II, III, V |
| 70 | *Sida rhombifolia* **L.** | Malvaceae | Yes |  |  | I, V |
| 71 | *Spilanthes costata* **Benth.** | Asteraceae | Yes |  |  | V |
| 72 | *Sporobolus pyramidalis* **P.Beauv.** | Poaceae | Yes |  |  | I, III, V |
| 73 | *Sterculia setigera* **Delile** | Malvaceae |  | Yes |  | I, II, III, V |
| 74 | *Synedrella nodiflora* **(L.) Gaertn.** | Asteraceae | Yes |  |  | I, III, IV, V |
| 75 | *Syzygium guineense* **(Willd.) DC.** | Myrtaceae |  | Yes |  | I, II, III |
| 76 | Tamarindus indica L. | Fabaceae |  | Yes |  | I, III |
| 77 | *Tephrosia bracteolata* **Guill. & Perr.** | Fabaceae | Yes |  | Yes | I, III, V |
| 78 | *Tephrosia elegans* **Schumach.** | Fabaceae | Yes |  | Yes | I, II, III, V |
| 79 | *Tephrosia flexuosa* **G.Don** | Fabaceae | Yes |  | Yes | III |
| 80 | *Theobroma cacao* **L.** | Malvaceae |  | Yes |  | I, III, IV |
| 81 | *Tridax procumbens* **L.** | Asteraceae | Yes |  |  | I, II, III, IV, V |
| 82 | *Triumfetta rhomboidea* **Jacq.** | Malvaceae | Yes |  |  | I, II, V |
| 83 | *Urena lobata* **L.** | Malvaceae | Yes |  |  | III, IV |
| 84 | *Vernonia amygdalina* **Delile (A–C).** | Asteraceae | Yes |  |  | III |
| 85 | Vitellaria paradoxa C.F.Gaertn. ssp. paradoxa | Sapotaceae |  | Yes |  | I, II, III, IV, V |
| 86 | *Vitex doniana* **Sweet** | Lamiaceae |  | Yes |  | I, II, III, V |
| 87 | *Xylopia aethiopica* **(Dunal) A. Rich.** | Annonaceae |  | Yes |  | III |
